# The duplicated cytochrome P450 CYP6P9a/b confers cross-resistance to a mitochondrial complex I inhibitor in the African malaria vector *Anopheles funestus*

**DOI:** 10.1186/s12864-025-11984-1

**Published:** 2025-09-26

**Authors:** Theofelix A. Tekoh, Leon M. J. Mugenzi, Benjamin Menze, Williams Tchapga, Murielle Wondji, Magellan Tchouakui, Graham Small, Charles S. Wondji

**Affiliations:** 1 Centre for Research in Infectious Diseases (CRID), P.O. Box 13501, Yaoundé, Cameroon; 2 Vector Biology Department, Liverpool School of Tropical Medicine, Pembroke Place, Liverpool, L35QA UK; 3 Syngenta Crop Protection, Werk Stein, Schaffhauserstrasse, Stein Switzerland; 4 Innovative Vector Control Consortium (IVCC), Liverpool, UK

**Keywords:** Pyrethroids, Cross-resistance, Complex I inhibitors, *An. funestus*, Metabolic resistance

## Abstract

Metabolic resistance to pyrethroids driven by cytochrome P450s is threatening malaria control interventions and may provide cross-resistance to insecticides with unrelated modes of action. Here, we show that cytochrome P450 genes *CYP6P9a/b* associated with pyrethroid resistance in *Anopheles funestus* also confer cross-resistance to a novel mitochondrial complex I inhibitor insecticide, code-named Sherlock. Using standard bioassays (CDC bottle bioassays, WHO cone bioassays, and WHO tunnel tests), the Sherlock and pyrethroid insecticides were tested against pyrethroid-resistant *An. gambiae* s.s. (Nkolondom, Cameroon) and *An. funestus* s.s. (Mibellon, Cameroon) and FUMOZ-R). Molecular assays (genotyping of P450 markers and qRT-PCR expression) were performed to investigate the underlying resistance mechanisms and cross-resistance in *An. funestus.* Field sampled strains of *An. gambiae* s.s. and *An. funestus* s.s. from Cameroon were fully susceptible to Sherlock, whereas moderate resistance was observed in the FUMOZ-R *An. funestus* strain. Genotypic analysis of hybrid mosquitoes demonstrated a correlation between pyrethroid-resistance markers and reduced susceptibility to Sherlock. Individuals carrying one *CYP6P9a*_R allele had significantly higher odds of surviving exposure to Sherlock compared to those lacking this allele, as evidenced by CDC bottle bioassays (1xDC: OR = 5.3, CI = 2.7–9.8, *p* < 0.0001; 5xDC: OR = 18.6, CI = 7.8–46.4, *p* < 0.0001)), cone bioassays (OR = 5.1, CI = 2.7–9.8, *p* < 0.0001), and tunnel tests (OR = 6.6, CI = 3.4–12.6 *p* < 0.0001). qRT-PCR analysis revealed elevated expression of *CYP6P9a* in surviving hybrid mosquitoes exposed to Sherlock and permethrin, as observed in CDC bottle bioassays (1xDC: FC = 24.7; 5xDC: FC = 45.6; permethrin: FC = 35.4) and cone bioassays (FC = 9.8; FC = 4.8, respectively). These findings were consistent with the patterns of *CYP6P9b* and the 6.5 kb insertion. The *L119F_GSTe2* pyrethroid resistance marker did not confer cross-resistance to Sherlock. These findings highlight the importance of considering cross-resistance patterns in the development and deployment of new insecticides for malaria vector control.

**Supplementary Information:**

The online version contains supplementary material available at 10.1186/s12864-025-11984-1.

## Background

Malaria remains a major debilitating disease that continues to plague the tropical world and was responsible for over 597,000 deaths and approximately 263 million cases in 2023 [1]. Efforts to reduce the malaria burden rely extensively on better diagnosis and treatment of malaria, and mosquito vector control involving the scale-up of interventions including indoor residual spraying (IRS), and insecticide-treated nets (ITNs), with many of the products including pyrethroid insecticides [1]. The scale-up of vector control interventions, especially ITNs, has been shown to result in a significant decrease in malaria cases across sub-Saharan Africa [2]. This, however, has been accompanied by increased insecticide selection pressure which has resulted in the evolution and spread of resistance mechanism to the pyrethroid class of insecticides [3–7]. There have been reports not only failures of pyrethroid-based control interventions but also of cross-resistance to other classes of insecticides with unrelated modes of action [8–12]. This has contributed to the stalling of the progress in reducing global malaria incidence and mortality rates against the target of an at least 90% reduction by 2030 [1, 13–15]. This has reinforced the need for malaria control programmes to implement resistance management strategies to preserve the effectiveness of current and future vector control tools [1].

One route to insecticide resistance management is the use of new insecticides with novel modes of action in addition to current insecticides, to sustain the effectiveness of ITN and IRS interventions [3]. Accordingly, the Global Plan for Insecticide Resistance Management (GPIRM) encouraged manufacturers or Product Development Partnerships such as the Innovative Vector Control Consortium (IVCC) to develop products containing insecticides novel to vector control to help manage resistance [13, 16]. IVCC, which was established in 2005, is integral to facilitating the development of novel insecticide products with the aspiration of eliminating malaria by managing resistance and, hence, maintaining the effectiveness of vector control tools [3, 17]. Many of the insecticides currently used in public health have been repurposed from crop protection or animal health. IVCC has also engaged with several agrochemical companies to generate a pipeline of novel insecticides for use in vector control products [3, 17]. As these novel vector control products exit the development pipeline, it is important that they be integrated into resistance management strategies [3, 17, 18]. The development of a new insecticide is a high-risk venture with high costs of development and long timelines from initial screening through to product launch [16]. It is, therefore, important to adopt strategies to detect and screen out compounds that can’t meet the target product profile before significant resources have been devoted to its development. One cost-efficient approach is to evaluate the efficacy of novel compounds of interest against pyrethroid resistant vector strains and wild populations to assess the risk of cross-resistance via the mechanisms of resistance they possess [16, 19]. This will help save time and money by stopping the further development of compounds already vulnerable to cross-resistance in some mosquito populations. Furthermore, growing reports of the escalation of resistance to pyrethroids might be associated with the selection of resistance mechanisms conferring a broader spectrum of cross-resistance to insecticides with other modes of action, including those still under development. Among known pyrethroid resistance mechanisms, metabolic resistance driven by upregulated cytochrome P450s (CYPs) is more likely to confer cross-resistance to a broad range of compounds owing to the ability of these enzymes to detoxify a range of substrates [20, 21]. This highlights the risk of cross-resistance to other insecticide classes, including Mitochondrial Complex I inhibitors [19]. Lees et al. (2020) used *in vitro* depletion assays to show that some Complex I inhibitor insecticides were highly susceptible to metabolism by pyrethroid resistance-associated *CYPs*, such as *CYP6M2* and *CYP6P3* in *An. gambiae* and *CYP6P9a* in *An. funestus*. Furthermore, transgenic mosquitoes overexpressing these *CYPs* exhibited reduced mortality, whereas piperonyl-butoxide (PBO)-synergist assays restored susceptibility, confirming the role of CYP-based metabolic cross-resistance [19]. Recent research has led to the detection of several DNA markers of resistance in detoxification enzymes in some malaria vector species, particularly *An. funestus* [9, 22–26]. These markers include those from the tandemly duplicated P450 genes *CYP6P9a/b* [24, 25] shown to confer pyrethroid resistance through allelic variation [27] and overexpression [28]. Furthermore, structural variants (SV) associated with enhanced expression of *CYPs* have also been detected including a 6.5 kb insertion in the promoter region of *CYP6P9a* [26], providing further methods to detect CYP-based resistance and assess its role in cross-resistance, as recently shown against carbamates [10].

This study aimed to establish a susceptibility profile of pyrethroid-resistant field collected and laboratory strains to a novel mitochondrial complex I inhibitor insecticide (Sherlock) under development by IVCC. The study evaluated the efficacy of Sherlock (active ingredient and in insecticide-treated nets) using CDC bottle assays, WHO cone bioassays and tunnel tests. The molecular basis of resistance and cross-resistance in pyrethroid-resistant strains generated from these assays was assessed using previously established DNA-based pyrethroid-resistance markers. Using CDC bottle assays, we demonstrated that the pyrethroid-resistant *An. funestus* strain FUMOZ-R laboratory strain was cross-resistant to the Sherlock insecticide, whereas pyrethroid-resistant *An. gambiae* s.s. and *An. funestus* s.s. strains sampled from populations in Cameroon were largely susceptible. We also observed a lower efficacy of Sherlock-impregnated ITNs against *An. funestus* s.s. laboratory strains. Efforts to delimit the molecular basis of this resistance demonstrated a strong correlation between the duplicated *CYP6P9a/b* genes associated with pyrethroid resistance; and the survival of *An. funestus* s.s. mosquitoes following exposure to Sherlock. This investigation not only elucidated the pivotal role of key pyrethroid resistance-associated *CYP* genes (*CYP6P9a/b*) in reducing susceptibility to the novel insecticide Sherlock but also highlighted the utility of integrating standard bioassays with molecular diagnostics as a cost-effective strategy to assess the biological efficacy and vulnerability to cross-resistance of insecticidal compounds during early-stage development.

## Methods

### Mosquito strains

*Field strains:* Two pyrethroid-resistant field strains collected in Cameroon were used to assess susceptibility to the mitochondrial complex I inhibitor, Sherlock: *An. gambiae* s.s. from Nkolondom (3^o^57′05″, 11 ^o^ 29′24″) [29] and *An. funestus* s.s. from Mibellon (6^o^46’N, 11^o^70’E) [12], a village in the Adamawa region (Mayo Banyo division). The larvae of *An. gambiae* s.s. (F_0_) were collected from breeding sites and brought to the Centre for Research in Infectious Diseases (CRID) laboratory in Yaoundé, where they were reared to the adult stage using Tetramin™ fish food. The F_0_ adults obtained were aspirated with a mouth aspirator into an adult cage and provided with a 10% sugar solution in preparation for use in susceptibility bioassays. Blood-fed indoor resting adult females of *An. funestus* s.s. (F_0_) were collected using an electric aspirator from homes (with consent from occupants) and put into paper cups. The collected adults were brought to the laboratory and maintained in cages for 5 days to allow for egg maturation. The fully gravid adult F_0_ females were subjected to forced oviposition [30] to obtain the F_1_ progeny that were then reared to the adult stage as previously described [30].

*Lab strains:* Two pyrethroid-resistant laboratory strains of *An. funestus* were colonised in CRID’s insectary; FUMOZ-R which was originally colonised from southern Mozambique and selected with 0.1% lambda-cyhalothrin to generate this highly pyrethroid resistant strain[31] and a hybrid strain generated from reciprocal crosses between the highly pyrethroid-resistant FUMOZ-R strain (possessing the *CYP6P9a*_R and *CYP6P9b*_R alleles)) and the fully insecticide susceptible laboratory FANG strain (possessing the *CYP6P9a*_S and *CYP6P9b*_S alleles) which was originally colonised in 2002 from Colueque, Southern Angola [31, 32]. To allow for substantial genetic recombination events, the obtained F_1_ progeny were intercrossed and reared until the fourth generation (F_4_) [26]. The F_4_ hybrid strain, comprising a mixture of different genetic recombinations, was used to establish an association between the different genotypes of the metabolic genes and the resistance phenotype (since these *CYP*s are fixed in the FUMOZ-R resistant strain).

### Susceptibility profile of mosquito strains against pyrethroids and Sherlock insecticides

Three standard bioassays were used to assess the susceptibility of field and laboratory strains to pyrethroids and to the Sherlock insecticide; CDC bottle bioassays, WHO cone bioassays, and WHO tunnel tests.

*CDC bottle assays:* To determine the susceptibility of field collected and laboratory strains to the Sherlock insecticide, a CDC bottle bioassay [33] was performed using bottles treated with the diagnostic concentration of Sherlock (1xDC; calculated as 3 × the LC_95_ for Sherlock in dose response bottle bioassays), and five times the diagnostic concentration (5xDC) for Sherlock (provided by IVCC). The strains were also exposed in bottles treated with permethrin (as a positive control) as well as a negative control bottle treated with only the solvent (acetone). Since the Sherlock insecticide bottles killed most of the mosquitoes exposed (except for the FUMOZ-R strain) using a 60 min exposure time, exposure time was reduced to 30 min to generate mosquito samples for genotyping and qRT-PCR (especially with hybrid strains having a reduced resistance background). Knockdown of mosquitoes was recorded 1-h post-exposure, and mortality was recorded 24 h post-exposure. Mosquitoes still alive at 24 h post-exposure were stored at −80 °C in RNAlater® (Thermo Fisher Scientific, UK) whilst dead mosquito samples were stored at −20 °C in 80% ethanol for downstream analyses. Bioassays were conducted under standard laboratory conditions of 25 ± 2 °C and 75 ± 10% relative humidity (RH). Where mortality in acetone-only negative control bottles was > 5%, mortalities of mosquitoes exposed to insecticide-treated bottles were corrected for control mortality using the Abbott’s formula [34].

### WHO cone bioassays

The bio-efficacy of a prototype 0.7% incorporated Sherlock net formulation (provided by IVCC) against the field and laboratory strains was evaluated as previously described [35]. The strains were also exposed in cones to samples of WHO Prequalification Unit Vector Control Product Assessment Team (PQT/VCP) listed deltamethrin-treated ITNs: PermaNet 2.0 (55 mg/m^2^ (± 25%)) and PermaNet 3.0 (85 mg/m^2^ (± 25%) with PBO synergist (750 mg/m^2^) on the roof added to the insecticide), and, an untreated net was used as a control. Two to five (2–5) days old unfed female mosquitoes were aspirated into paper cups (10 mosquitoes per cup) and tested against 30 cm × 30 cm cut pieces of each net using plastic cones. After 3 min of exposure, mosquitoes were aspirated out of the cone, transferred back into paper cups and fed with cotton wool soaked with 10% sugar solution. The percentage mortality was recorded 24 h post-exposure. All net types were also tested against the insecticide-susceptible *An. gambiae* s.s. Kisumu strain. Bioassays were conducted under standard laboratory conditions of 25 ± 2 °C and 75 ± 10% RH and the samples were preserved as described above. Mortalities of mosquitoes exposed to insecticide-treated net samples were corrected for mortality with untreated net samples using the Abbott’s formula [34].

### WHO tunnel tests

The WHO tunnel test was used to further evaluate the bio-efficacy of the different insecticide-treated nets against the field strain (Mibellon) and the laboratory strains (FUMOZ-R, FUMOZ-R/FANG F_4_ hybrids, and Kisumu) by evaluating the mortality and ability of host-seeking mosquitoes to blood feed on an animal bait [35]. This test was conducted using a square tunnel made of glass panels with a size of 60 × 25 × 25 cm (L x W x H), following WHO guidelines [35] The tunnel has two compartments, one with the bait and the other in which the host-seeking mosquitoes are released to voluntarily cross a bed net barrier before reaching the animal bait. Samples of the different bed nets (0.7% incorporated Sherlock net, PermaNet 2.0 net, PermaNet 3.0 net (roof), and an insecticide-free bed net) were cut into pieces measuring 30 cm × 30 cm and nine holes (1 cm in diameter) were cut at an equidistance of 5 cm from the border, to allow the passage of mosquitoes. Tunnel tests bioassays were conducted under standard laboratory conditions of 27 ± 2 °C and 75 ± 10% RH. One hundred (100) 5–8 days old unfed female mosquitoes were aspirated into paper cups before the tunnel apparatus was set up. They were briefly sugar-starved before being released into the tunnel through the mosquito-release compartment. The mosquitoes were exposed to the cut sample of bed net overnight for 14 h (introduced at 19:00 h in the evening and collected at 09:00 h the following morning) before mosquito knockdown was recorded. Mosquitoes were aspirated from each chamber of the tunnel into paper cups. They were fed with cotton wool soaked in a 10% sugar solution; the mortality was recorded 24 h post-exposure. The live and dead mosquito samples were preserved as described above for downstream analyses. Mortalities of mosquitoes exposed in tunnels to insecticide-treated net samples were corrected for mortality with untreated net samples (mortality ≤ 20%) using Abbott’s formula[34].

### Genotyping of pyrethroid-resistant markers; the L119F_*GSTe2* DDT/pyrethroid-resistant marker in the field samples, and, 6.5 kb SV, *CYP6P9a* and *CYP6P9b* in the hybrid samples

Genomic DNA was extracted from whole mosquitoes as previously described [36]. Since the *CYP*-resistance markers are absent in the field populations from Cameroon [26], the *L119F_GSTe2* DDT/pyrethroid-resistance marker was genotyped in bioassay-generated field samples using an allele-specific PCR assay previously described by [37]. The allele-specific primers discriminate between mosquitoes with either the *L119-* or the *119F-GSTe2* alleles. While all individuals present a common band at 849 bp, *119F/F* and *L/L119* individuals present specific bands at 523 bp and 312 bp, respectively. A PCR reaction for gene amplification was set up using Kapa Taq polymerase (Thermo Fisher). The thermal cycler conditions were set to an initial denaturation at 95 °C (for 5 min), 35 successive cycles of 94 °C (30secs), 58 °C (1 min) and 72 °C (45 s) and a final extension at 72 °C (2 min).

The *CYP*-resistance markers were genotyped in FUMOZ-R/FANG F_4_ hybrid mosquitoes to establish whether there was a correlation between these markers and the mosquitoes’ ability to survive insecticide exposure. A PCR amplification followed by a restriction fragment length polymorphism (PCR–RFLP) assay was used to distinguish the R- and S- alleles of *CYP6P9a* [24] and *CYP6P9b* [25]. An amplified fragment of the target genes was digested with a restriction enzyme to produce separate bands of the R allele (*CYP6P9a*: 350 bp and 100 bp; *CYP6P9b*: 550 bp) and the S -allele (*CYP6P9a*: 450 bp; *CYP6P9b*: 400 bp and 150 bp). The thermal cycler conditions were set as follows; initial denaturation at 95 °C for 5 min, 35 cycles of 94 °C (30 s), 58 °C (1 min) and 72 °C (45 s), and a final extension at 72 °C for 10 min. For the 6.5 kb SV, allele-specific primers previously designed by Mugenzi et al., [26] were used to distinguish between mosquitoes with (596 bp) and without (266 bp) the SV. The PCR products were resolved on a 1.5% agarose gel and then visualized in an Enduro^TM^GDS gel documentation system.

### The expression levels of metabolic genes

The level of expression of the pyrethroid detoxification genes was assessed by qRT-PCR to establish a link between gene expression and cross-resistance to Sherlock in hybrid lab and field (Mibellon) strains of *An. funestus*. RNA was extracted from live mosquito samples stored in RNAlater® using the Arcturus™PicoPure™ RNA isolation kit (ThermoFisher Scientific, USA). The RNA extracts were reverse transcribed into complementary DNA (cDNA) using the SuperScript^TM^III Reverse Transcriptase cDNA synthesis kit (Invitrogen). A qRT-PCR was performed to compare the gene expression level in exposed, unexposed, and susceptible control groups using the Agilent MX3005P thermocycler (Agilent) as previously described [28]. The obtained CT values were then analysed by the comparative C(t) (2^−ΔΔCT^) method while being normalized to 2 housekeeping genes; *Actin 5C* (AFUN006819-RA) and *RSP7* (AFUN007153-RA) [38].

### Statistical analysis

All the statistical analyses and graphical illustrations of the results were performed using GraphPad Prism 8.0.2. Fisher’s exact test was performed to assess any significant difference in the distribution of proportions for genotype contingency between the dead and alive mosquito groups. The odds ratio was used to measure the strength of the association between a resistance marker and the ability to survive insecticide exposure. The Student’s t-test was performed to evaluate any significant difference between the means of the data obtained for the qPCR expression analyses. The results for the alpha values were considered significant if the *p*-value was less than 0.05.

## Results

### Susceptibility bioassays against strains

#### CDC bottle assays

**Fig. 1:**
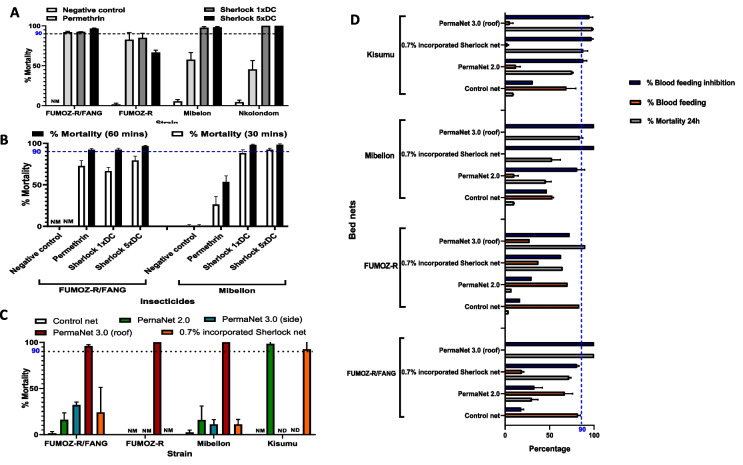
Insecticide susceptibility profile of mosquito strains. **A** CDC bottle bioassay showing mortality of FUMOZ-R/FANG F_4_ hybrids, pyrethroid-resistant FUMOZ-R laboratory strain and sampled pyrethroid-resistant field strains of *An. funestus* s.s. (Mibellon) and *An. gambiae* s.s. (Nkolondom) **B**) CDC bottle bioassays showing the mortalities of both FUMOZ-R/FANG F_4_ hybrids and sampled pyrethroid-resistant *An. funestus* s.s. field strain (Mibellon) to permethrin and Sherlock doses after 30 min and 60 min exposure. **C** WHO cone bioassay showing mortality of FUMOZ-R/FANG F_4_ hybrids, pyrethroid-resistant laboratory strain FUMOZ-R, sampled pyrethroid-resistant field strains of *An. funestus* s.s. (Mibellon) and the insecticide susceptible laboratory strain KISUMU **D**) WHO tunnel test showing mortality, blood feeding and inhibition of the FUMOZ-R/FANG F_4_ hybrids, FUMOZ-R pyrethroid-resistant laboratory strain, field collected *An. funestus* s.s. (Mibellon) and KISUMU susceptible strain to different bed nets (The error bars indicate the standard error of the mean). NM: No mortality, ND: No Data

The *An. gambiae* s.s. strain collected from Nkolondom was resistant to permethrin (mortality 45.5 ± 10.98%) but was fully susceptible to both Sherlock insecticide doses (1xDC and 5xDC) 24 h post-exposure (Fig. 1A). Similarly, field collected *An. funestus* s.s. from Mibellon were resistant to permethrin (mortality 55 ± 7.2%) but were almost fully susceptible to Sherlock 1xDC (mortality 98 ± 0.9%) and Sherlock 5xDC (mortality 98.3 ± 1.1%). The FUMOZ-R laboratory strain was also resistant to permethrin (mortality 82.6 ± 9.1%), but, unlike the field strains from Cameroon, it showed cross-resistance to Sherlock with lower mortality with both the 1xDC (85 ± 6.1%) and 5xDC (66.6 ± 2.96%) (Fig. 1A). These findings suggest that the mosquitoes exhibit high-intensity cross-resistance to the Sherlock insecticide. The FUMOZ-R/FANG F_4_ hybrids showed possible resistance to permethrin, with a mortality rate of 92 ± 1.1%. Similarly, mortality was 93 ± 0.8% with the Sherlock 1xDC dose and 97 ± 0.97% with the 5xDC dose, compared to the negative control (solvent only) with no mortality (Fig. 1A). These results suggest that the resistance mechanisms reducing susceptibility to Sherlock are present in *An. funestus* s.s. FUMOZ-R but absent in the *An. gambiae* s.s. and *An. funestus* s.s. populations in Cameroon.

To facilitate further molecular analyses, such as genotyping and qRT-PCR, the exposure time to the different insecticides was shortened to 30 min for a subset of FUMOZ-R/FANG F_4_ hybrids and field (Mibellon) *An. funestus* strains. With this reduced exposure, lower mortality rates were observed for permethrin, Sherlock 1xDC, and Sherlock 5xDC. The mortality rates were as follows; for Mibellon, 29.3 ± 10%, 88.5 ± 4.1% and 92.3 ± 1.6%, respectively, and for FUMOZ-R/FANG F_4_ hybrids 73 ± 6.2%, 67 ± 4.6%, and 80 ± 5.1%, respectively (Fig. 1B, Table in Sup. 1a).

#### WHO cone bioassays

Cone bioassays with *An. funestus* sampled from the population in Mibellon presented low mortality rates 24 h post-exposure with percentage mortality rates 11 ± 3.1%, 17 ± 5.5%, and 9 ± 3.1% against 0.7% incorporated Sherlock net, PermaNet 2.0, and PermaNet 3.0 (side), respectively, when compared to the control net (2 ± 1.2%). However, they all died (100% mortality) when exposed to the PBO-containing compartment of PermaNet 3.0 (roof) (Fig. 1C). The bed nets showed low efficacy (0% mortality) against the FUMOZ-R strain, except for PermaNet 3.0 (roof) (100% mortality). All bed nets also showed low efficacy against the FUMOZ-R/FANG F_4_ hybrids with percentage mortalities of 17 ± 3.5%, 21 ± 4.9%, and 32 ± 4.6% corresponding to PermaNet 2.0, Sherlock and PermaNet 3.0 (side), respectively, compared to the untreated net (2 ± 0.9%). The PBO-containing PermaNet 3.0 (roof) killed 96 ± 4.5% of the exposed F_4_ hybrids (Fig. 1C). This suggests a major role of *CYP* genes in conferring pyrethroid resistance in this strain. WHO cone bioassays were not performed with the *An. gambiae* field strain since we did not find any resistance to Sherlock in the bottle assays.

#### WHO tunnel tests

*Mortalities:* Overall, mortalities in the tunnel tests were significantly higher than those in the cone tests. Similarly to the results from WHO cone bioassays, PermaNet 2.0, 0.7% incorporated Sherlock and PermaNet 3.0 (roof) showed a lower efficacy against the Mibellon strain 24 h post-exposure with mortalities of 45.8 ± 6%, 52.81 ± 9.3%, and 83.8 ± 3.7%, respectively. Similarly, the FUMOZ-R and hybrid strains had low mortality rates against PermaNet 2.0 and Sherlock net but not PermaNet 3.0 net (roof) with percentage mortalities: for FUMOZ-R mortalities were 6.9%, 64.7% and 90.1%, respectively. The percentage mortalities for the FUMOZ-R/FANG F_4_ hybrids to these nets was 30 ± 7%, 72 ± 2% and 100 ± 0%, corresponding to PermaNet 2.0, Sherlock net and PermaNet 3.0 (roof), respectively. These percentage mortalities were computed using only the unfed mosquitoes, to remove possible bias and artifacts resulting from blood-feeding stress. While PermaNet 3.0 (roof with PBO) induced high mortality rates against the susceptible Kisumu strain (98.3 ± 0.8%), mortality with PermaNet 2.0 and Sherlock nets was lower (75.3 ± 1.3%, and 87.9 ± 4.9%, respectively) (Fig. 1D).

*Blood feeding and blood-feeding inhibition:* Only 10 ± 4.6% of the field strain (Mibellon) successfully blood-fed through the PermaNet 2.0 bed net, whereas blood feeding was completely inhibited by both the Sherlock and PermaNet 3.0 (roof) nets. This finding indicates that Sherlock meets the WHO criteria, as it performs as well as or better than the reference ITN (PermaNet 2.0) in preventing blood-feeding and inducing mortality [35]. In contrast, a greater proportion of Mibellon mosquitoes (53 ± 1.4%) were blood-fed when exposed in tunnels to the untreated control net.

For the FUMOZ-R strain, the blood-feeding rates were 70.3% for PermaNet 2.0, 37.3% for Sherlock, and 27.5% for PermaNet 3.0 (roof), whilst the blood-feeding rate was 83% for the untreated control net. Notably, all treated bed nets effectively reduced blood-feeding in FUMOZ-R, with inhibition rates of 29.7% (PermaNet 2.0), 62.7% (Sherlock), and 72.5% (PermaNet 3.0 (roof)), relative to the control net (16.8%) (Fig. 1D).

A similar trend was observed with the FUMOZ-R/FANG F_4_ hybrids, as more mosquitoes were able to cross through the untreated net barrier and blood feed (82 ± 2.7%), than those exposed to insecticide-impregnated bed nets (PermaNet 2.0 (67 ± 9%) and Sherlock (19 ± 2%)). Moreover, the insecticide containing bed nets better inhibited the blood-feeding ability of FUMOZ-R/FANG F_4_ hybrid mosquitoes (33 ± 9% (PermaNet 2.0) and 81 ± 2% (Sherlock net)) than did the untreated bed net (18 ± 2.7%). No FUMOZ-R/FANG F_4_ hybrids were able to blood feed through PermaNet 3.0 (roof) (Fig. 1D). All insecticide-treated nets inhibited > 80% of blood-feeding in the susceptible Kisumu strain, suggesting that resistance mechanisms present in the other mosquito strains contributed to higher blood feeding success despite insecticide exposure.

### Genotyping of pyrethroid-resistance markers; 6.5 kb SV, *CYP6P9a* and *CYP6P9b* in bioassay-generated samples

#### CDC bottle bioassays

The genotyping of 6.5 kb SV, *CYP6P9a*, and *CYP6P9b* resistance markers compared FUMOZ-R/FANG F_4_ hybrid samples from permethrin, Sherlock 1xDC, and Sherlock 5xDC exposures between survivors and non-survivors.

#### Correlation between the 6.5 kb SV and the bio-efficacy of the permethrin and Sherlock insecticide bottles

Comparative genotyping of the 6.5 kb SV in the dead and live mosquito samples revealed a strong correlation between the presence of the 6.5 kb SV and mosquito survival following insecticide exposure. The hybrids homozygous for the 6.5 kb SV (SV^+^/SV^+^) genotype had a greater chance of surviving exposure to the different insecticides than did the heterozygous individuals (SV^+^/SV^−^) (permethrin (OR = 23.6; CI = 10.1–53.7; *p* < 0.0001), Sherlock 1xDC (OR = 7.96; CI = 3.9–16.0; *p* < 0.0001), Sherlock 5xDC (OR = 17.4; CI = 8.2–36.8; *p* < 0.0001)) and those homozygous for the absence of the SV (SV^−^/SV^−^) (permethrin (OR = 30.9; CI = 9–100.7; *p* < 0.0001), Sherlock 1xDC (OR = Inf.; CI = 15.3-Inf.; *p* < 0.0001) and Sherlock 5xDC (OR = Inf.; CI = 31.9-Inf., *p* < 0.0001)) (Table 1). This suggests that FUMOZ-R/FANG F_4_ hybrids with two SV^+^ alleles have an added advantage in surviving exposure to these two insecticides (Figures of frequencies in Sup.1b).

**Table 1:** Correlation between the genotypes of the 6.5 kb SV, *CYP6P9a* and *CYP6P9b* and the ability of FUMOZ-R/FANG F_4_ hybrids to survive exposure to Permethrin, Sherlock 1xDC, and Sherlock 5xDC bottles ('Inf.’ Stands for ‘Infinite values’)

		**OR**	**CI**	**P value**	**OR**	**CIP value**		**OR**	**CI**	**P value**
			***6.5 kb***			***CYP6P9a***			***CYP6P9b***	
**Permethrin**	**RR vs SS**	30.9	9.0–100.7	< 0.0001	44.6	11.2- 149.0	< 0.0001	23.5	6.3–78.3	< 0.0001
	**RS vs SS**	1.31	0.3–4.9	> 0.9999	2.0	0.6–6.98	0.3919	2.0	0.6–6.9	0.389
	**RR vs RS**	23.6	10.1–53.7	< 0.0001	21.9	9.9–47.9	< 0.0001	11.7	5.7–23.7	< 0.0001
	**R vs S**	13.8	5.8–34.3	< 0.0001	8.96	4.4–18.0	< 0.0001	7.05	3.4–14.2	< 0.0001
**Sherlock** **1xDC**	**RR vs SS**	Inf	15.3-Inf	< 0.0001	Inf	17.3-Inf	< 0.0001	Inf	19.7–Inf	< 0.0001
	**RS vs SS**	Inf	1.7-Inf	0.0088	Inf	4.1-Inf	< 0.0001	Inf	2.8-Inf	0.0008
	**RR vs RS**	7.96	3.9–16.0	< 0.0001	4.3	2.2–7.8	< 0.0001	7.8	3.95–14.9	< 0.0001
	**R vs S**	9.8	4.4–20.8	< 0.0001	5.3	2.7 – 9.8	< 0.0001	5.7	2.9–10.6	< 0.0001
**Sherlock** **5xDC**	**RR vs SS**	Inf	31.9-Inf	< 0.0001	Inf	45.0-Inf	< 0.0001	Inf	86.2-Inf	< 0.0001
	**RS vs SS**	Inf	1.9-Inf	0.0058	Inf	2.0-Inf	0.0045	Inf	2.7-Inf	0.0009
	**RR vs RS**	17.4	8.2–36.8	< 0.0001	22.3	9.7–46.6	< 0.0001	34.8	13.4–86.1	< 0.0001
	**R vs S**	11	5.0–22.4	< 0.0001	18.6	7.8–46.4	< 0.0001	11.5	5.5–23.3	< 0.0001

Similarly, mosquitoes with SV^+^/SV^−^ genotype were significantly more likely to survive Sherlock exposure than were SV^−^/SV^−^ at both the 1xDC (OR = Inf.; CI = 1.7-Inf.; *p* = 0.0088) and 5xDC (OR = Inf.; CI = 1.9-Inf.; *p* = 0.0058). However, no significant difference in survival was detected between SV^+^/SV^−^ and SV^−^/SV^−^ mosquitoes following permethrin exposure (OR = 1.3; CI = 0.3–4.9; *p* > 0.9999). Overall mosquitoes carrying the (SV^+^) insertion had a significantly greater survival chance against permethrin (OR = 13.8, CI = 5.8 to 34.3, p < 0.0001), the Sherlock 1xDC (OR = 9.8, CI = 4.4 to 20.8, p < 0.0001), and Sherlock 5xDC (OR = 11.5, CI = 5.0–22.4, p < 0.0001) than those with SV^−^ (Table 1).

#### Correlation between CYP6P9a and the bio-efficacy of the permethrin and Sherlock insecticide bottles

The genotyping of the *CYP6P9a* resistance marker in both surviving and dead mosquito samples revealed a similar pattern. Hybrids homozygous for the resistance allele (RR) had a significantly greater survival ability across all insecticide exposures compared to heterozygous (RS) individuals (permethrin (OR = 21.9; CI = 9.9–47.9; *p* < 0.0001), Sherlock 1xDC (OR = 4.3; CI = 2.2–7.8; *p* < 0.0001), and Sherlock 5xDC (OR = 22.3; CI = 9.7–46.6; *p* < 0.0001)). Similarly, RR hybrids had a significantly greater survival advantage over homozygous susceptible (SS) mosquitoes (permethrin (OR = 44.6; CI = 11.2–149; *p* < 0.0001), Sherlock 1xDC (OR = Inf.; CI = 17.3-Inf.; *p* < 0.0001), Sherlock 5xDC (OR = Inf.; CI = 45.0-Inf.; *p* < 0.0001)) (Table 1). These findings suggest that carrying two resistance alleles provide an added survival advantage against exposure to these two insecticides (Figures of frequencies in Sup. 1c).

Furthermore, hybrids with the RS genotype also survived insecticide exposure better than those with the SS genotype (Sherlock 1xDC (OR = Inf.; CI = 4.1-Inf.; *p* < 0.0001), Sherlock 5xDC (OR = Inf.; CI = 2.0-Inf.; *p* = 0.0045)) suggesting that having one allele of this gene is sufficient to confer some level of cross-resistance to the Sherlock insecticide. Similarly to the 6.5 kb SV, no significant difference in survival was observed between the RS and SS genotypes following permethrin exposure (OR = 2.0; CI = 0.6–6.98; *p* = 0.3919). However, the R allele in general had a greater chance of surviving insecticide exposure than did the S allele (permethrin (OR = 8.96; CI = 4.4–18.0; *p* < 0.0001), Sherlock 1xDC (OR = 5.3; CI = 2.7–9.8; *p* < 0.0001) and Sherlock 5xDC (OR = 18.6; CI = 7.8–46.4, *p* < 0.0001)) (Table 1).

#### Correlation between CYP6P9b and the bio-efficacy of the permethrin and Sherlock insecticide bottles

A similar trend was observed after genotyping *CYP6P9b* in the dead and alive samples which was strongly correlated with the reduced efficacy of the different insecticides. Compared with those with the RS genotype, hybrids with the RR genotype were more likely to survive exposure to the different insecticides (permethrin (OR = 11.7; CI = 5.7–23.7; *p* < 0.0001), 1 × DC (OR = 7.8; CI = 3.95–14.9; *p* < 0.0001), 5xDC (OR = 34.8; CI = 18.4–86.1; *p* < 0.0001)) and those with the SS genotype (permethrin (OR = 23.5; CI = 6.3–78.3; *p* < 0.0001), 1xDC (OR = Inf.; CI = 19.7-Inf.; *p* < 0.0001), 5xDC (OR = Inf; CI = 86.2-Inf.; *p* < 0.0001)) (Table 1). This suggests an added survival advantage for mosquitoes possessing two R alleles. Hybrids having the RS genotype were more likely to survive compared to those in the SS genotype (1xDC (OR = Inf.; CI = 2.8-Inf.; *p* = 0.0008), 5xDC (OR = Inf.; CI = 2.7-Inf.; *p* = 0.0009)). For permethrin exposed, there was no significant difference between the RS and SS genotypes (OR = 2.0; CI = 0.6–6.9; *p* = 0.389). Overall, the R allele could better survive exposure compared to the susceptible allele (permethrin (OR = 7.1; CI = 3.4–14.2; *p* < 0.0001), 1xDC (OR = 5.7; CI = 2.9–10.6; *p* < 0.0001) and 5xDC (OR = 11.5; CI = 5.5–23.3, *p* < 0.0001)) (Table 1, Figures of frequencies in Sup. 1d).

#### The 6.5 kb SV, CYP6P9a and CYP6P9b combined to reduce insecticide efficacy

**Fig. 2:**
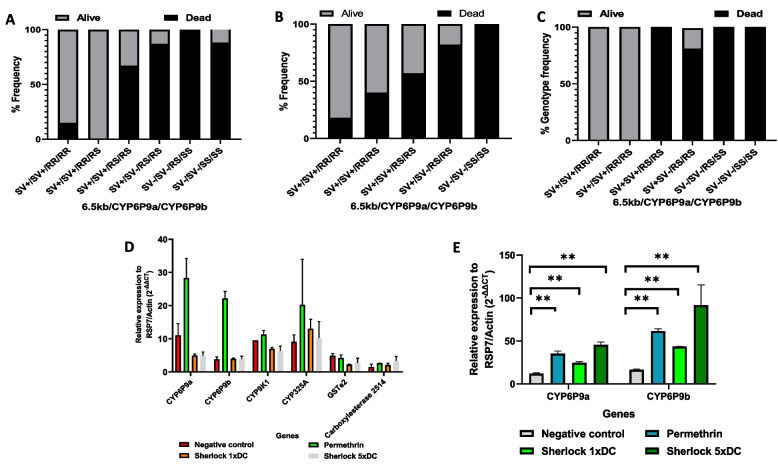
Genotype results for CDC bottle bioassay. The association of the combined three genotypes of the 6.5 kb SV, CYP6P9a_R and CYP6P9b_R alleles with the resistance phenotype. FUMOZ-R/FANG F_4_ hybrids exposed to **A**) Permethrin-impregnated bottles, **B**) Sherlock 1xDC, and **C**) Sherlock 5xDC. QRT-PCR analyses of metabolic genes in **D**) FUMOZ-R/FANG F_4_ hybrids, and E) Mibellon field-collected strains alive after bottle bioassays

Combining the three markers conferred a survival advantage, which was most pronounced at the highest dose of the Sherlock insecticide (5xDC). Overall, mosquitoes possessing triple homozygous resistance alleles at the three different loci (SV^+^/SV^+^/RR/RR) had the greatest chance of surviving exposure to the different insecticides (Fig. 2 A, B and C), compared to mosquitoes triple homozygous without any resistant allele (SV-/SV-/SS/SS) (Permethrin (OR = 38.4; CI = 17.1–79.7; *p* < 0.0001), Sherlock 1xDC (OR = Inf.; CI = 120.8-Inf.; *p* < 0.0001), and Sherlock 5xDC (OR = Inf.; CI = 1980-Inf.; *p* < 0.0001)). These results support the additive effect of resistance as possessing 6 resistant alleles significantly reduces the efficacy of pyrethroids and an overall greater level of cross-resistance to sherlock (Table of significance in Sup. 1e).

### WHO cone bioassays

#### Genotyping of the L119F_GSTe2 resistance marker in the filed strains

Genotyping analysis revealed no significant association between the L119F-GSTe2 marker and survival after exposure to the Sherlock net, although all mosquitoes with the RR genotype survived (100%). However, when examining mosquitoes exposed to PermaNet 2.0, a significant difference in genotype distribution was observed between the dead and alive groups for this marker (*X*^*2*^ = 39.56, *p* < 0.0001) (Figure in Sup. 2a). In comparison to the RS genotype, the RR genotype significantly increased the likelihood of survival (OR = Inf.; CI = 1.49-Inf.; p = 0.0159), as did the SS genotype (OR = Inf.; CI = 1.96-Inf.; p = 0.0059). However, no significant difference in survival was observed between the RS and SS genotypes (OR = 1.23; CI = 0.67–2.25; p = 0.5454) (Table of significance in Sup. 2b).

#### Correlation between the 6.5 kb SV and the bio-efficacy of pyrethroid and Sherlock bed net

**Fig. 3:**
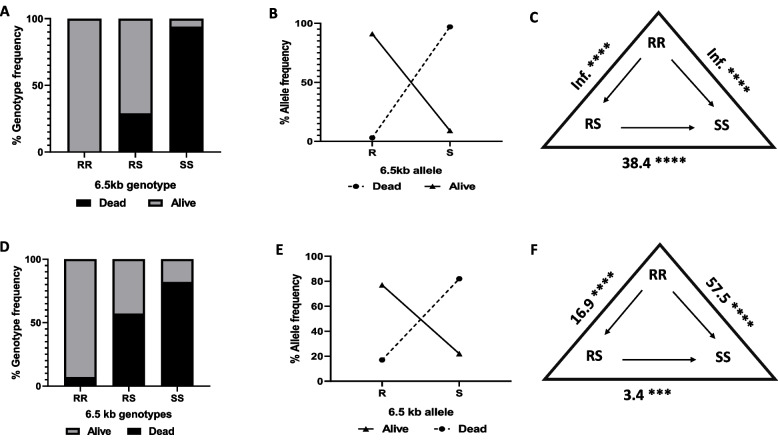
Genotype and allele frequencies of the 6.5 kb SV in WHO cone assays. Distribution of the 6.5 kb SV genotypes between the dead and alive F_4_ hybrids exposed to **A**) PermaNet 2.0. **D** Sherlock net. Allele frequencies of 6.5 kb *SV* between the dead and alive FUMOZ-R/FANG F_4_ hybrids exposed to **B**) PermaNet 2.0 and **E**) Sherlock net. Estimation of odds ratio (OR) and associated significance between different genotypes and the ability to survive exposure to C) PermaNet 2.0 and F) Sherlock net. The level of significance is indicated as *P < 0.05, **P < 0.01, ***P < 0.001 as estimated by Fisher’s exact test, the arrows in the triangle indicate the direction of OR estimation and ‘Inf.’ Stands for infinity

Genotyping the 6.5 kb SV between dead and alive FUMOZ-R/FANG F_4_ hybrids revealed a significant genotype distribution (PermaNet 2.0 (*X*^*2*^ = 191.6, *p* < 0.0001) and Sherlock net (*X*^*2*^ = 116.7, *p* < 0.0001)), indicating a strong association with bed net bio-efficacy (Table 2, Fig. 3).

**Table 2:** Correlation between genotypes of the 6.5 kb SV, *CYP6P9a* and *CYP6P9b* and the ability of FUMOZ-R/FANG F_4_ hybrids to survive exposure to 0.7% incorporated Sherlock net and PermaNet 2.0 (WHO cone bioassays) ('Inf.’ Stands for ‘Infinite values’)

		**OR**	**CI**	**P value**	**OR**	**CI**	**P value**	**OR**	**CI P value**	
			***6.5 kb***			***CYP6P9a***			***CYP6P9b***	
**0.7% incorporated Sherlock net**	**RR vs SS**	57.5	21.3–156.4	< 0.0001	22.6	6.8- 65.2	< 0.0001	72.6	16.6–315	< 0.0001
	**RS vs SS**	3.4	1.5–8.1	0.0046	9.7	4.8–19.6	< 0.0001	5.1	2.5–9.9	< 0.0001
	**RR vs RS**	16.9	5.8–47.3	< 0.0001	2.3	0.8–6.7	0.2067	14.2	3.4–62.3	< 0.0001
	**R vs S**	16.2	8.1–32.1	< 0.0001	5.1	2.7–9.8	< 0.0001	7.13	3.7–13.8	< 0.0001
**PermaNet 2.0**	**RR vs SS**	Inf	318.8-Inf	< 0.0001	134.3	48–351.4	< 0.0001	96.2	33.96–256.2	< 0.0001
	**RS vs SS**	38.4	15.5–91.7	< 0.0001	326.9	86.3–1059	< 0.0001	Inf	156.1-Inf	< 0.0001
	**RR vs RS**	Inf	11.56-Inf	< 0.0001	0.4	0.1–1.6	0.3311	0	0–0.7	0.0289
	**R vs S**	326.9	86.3–1059	< 0.0001	36	12.4–96.02	< 0.0001	50.6	15.96–158.5	< 0.0001

With PermaNet 2.0, hybrids homozygous for the 6.5 kb (SV^+^/SV^+^) had a significantly higher survival odds than heterozygotes (SV^+^/SV^−^) (OR = Inf.; CI = 11.56-Inf. *p* < 0.0001) and those without the SV (SV^−^/SV^−^) (OR = Inf.; CI = 318.8-Inf. *p* < 0.0001). Similarly, SV^+^/SV^−^ hybrids had a significantly greater chance of survival than SV^−^/SV^−^ hybrids (OR = 38.4; CI = 15.5–91.7; *p* < 0.0001). Overall, the SV^+^ allele was strongly associated with survival compared with the SV^−^ allele (OR = 326.9; CI = 86.25–1059; *p* < 0.0001) (Fig. 3 B).

A similar pattern was observed with Sherlock net exposed hybrids. Hybrids with the SV^+^/SV^+^ genotype showed increased survival to exposure compared to those with the SV^+^/SV^−^ genotype (OR = 16.9; CI = 5.8–47.3; *p* < 0.0001), and those with the SV^−^/SV^−^ genotype (OR = 57.5; CI = 21.3–156.4; *p* < 0.0001) (Figs. 3D and F). This finding suggests an added survival advantage when the two alleles are present. Additionally, the survival odds were significantly greater for the SV^+^/SV^−^ hybrids than for the SV^−^/SV^−^ hybrids (OR = 3.4; CI = 1.5–8.1; *p* = 0.0046). Overall, the SV^+^ allele was more associated with the resistance phenotype than the SV^−^ allele (OR = 16.15; CI = 8.066–32.07; *p* < 0.0001) (Table 2, Fig. 3E).

#### Correlation between CYP6P9a and the bio-efficacy of pyrethroid and Sherlock bed nets

**Fig. 4:**
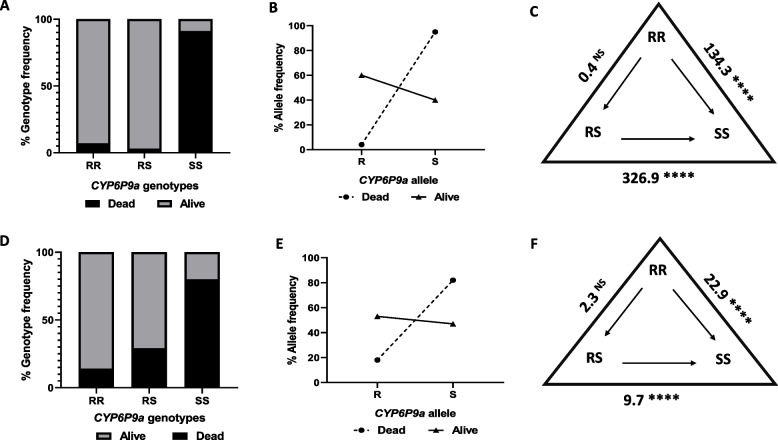
Genotype and allele frequencies of *CYP6P9a* in WHO cone assays. Distribution of the *CYP6P9a* genotypes between the dead and alive FUMOZ-R/FANG F_4_ hybrids exposed to **A**) PermaNet 2.0. **D**) Sherlock net Allele frequencies of *CYP6P9a* between the dead and alive FUMOZ-R/FANG F_4_ hybrids exposed to **B**) PermaNet 2.0 and **E**) Sherlock net. Estimation of odds ratio (OR) and associated significance between different genotypes and the ability to survive exposure to C) PermaNet 2.0 and **F**) Sherlock net. The level of significance is indicated as *P < 0.05, **P < 0.01, ***P < 0.001 as estimated by Fisher’s exact test, the arrows in the triangle indicate the direction of OR estimation and ‘Inf.’ Stands for infinity

There was a similar observation with the *CYP6P9a* resistance marker when comparing dead and alive FUMOZ-R/FANG F_4_ hybrid samples generated from PermaNet 2.0 exposure. The genotypes were significantly distributed between the dead and alive groups (*X*^*2*^ = 221.1, *p* < 0.0001). No significant difference in survival was observed between the homozygous resistant genotype (RR) and the heterozygous resistant genotype (RS) to PermaNet 2.0 exposure (OR = 0.4; CI = 0.1–1.6; *p* = 0.3311) (Table 2, Figs. 4A and C). However, hybrids with the RR genotype had a greater chance of survival than hybrids with the homozygous susceptible (SS) genotype (OR = 134.3; CI = 48.01–351.4; *p* < 0.0001). Similarly, the RS hybrids revealed significant increased survival odds to PermaNet 2.0 exposure compared to the SS hybrids (OR = 326.9, CI = 85.3–1059, *p* < 0.0001) implying that one resistant allele of the *CYP6P9a* is sufficient to confer resistance against PermaNet 2.0. In general, the R allele had a greater chance of surviving PermaNet 2.0 exposure than the S allele (OR = 36, CI = 12.35–96.02, *p* < 0.0001) (Table 2, Fig. 4B).

A similar trend was observed in FUMOZ-R/FANG F_4_hybrids exposed to the Sherlock net. There was a significant distribution of genotypes between dead and alive mosquito samples (*X*^*2*^ = 98.97, *p* < 0.0001) (Figure D). Compared with those with the SS genotype, hybrids with the RR genotype survived Sherlock net exposure better (OR = 22.6; CI = 6.8–65.2; *p* < 0.0001) (Fig. 4F). Like PermaNet 2.0 exposure, no significant difference in survival ability was found between individuals with the RR and the RS genotype and the survival of Sherlock net exposure (OR = 2.3; CI = 0.8–6.7; *p* = 0.2067). However, hybrids with the RS genotype had a greater chance of surviving Sherlock net exposure than those with the SS genotype (OR = 9.7; CI = 4.8–19.6; *p* < 0.0001), suggesting that one allele is sufficient to confer cross-resistance to the Sherlock insecticide when incorporated into a bed net. Overall, the R allele was more associated with survival than the S allele (OR = 5.14; CI = 2.67–9.819; *p* < 0.0001) (Table 2, Fig. 4E).

#### Correlation between CYP6P9b and the bio-efficacy of PermaNet 2.0 and Sherlock bed net

**Fig. 5:**
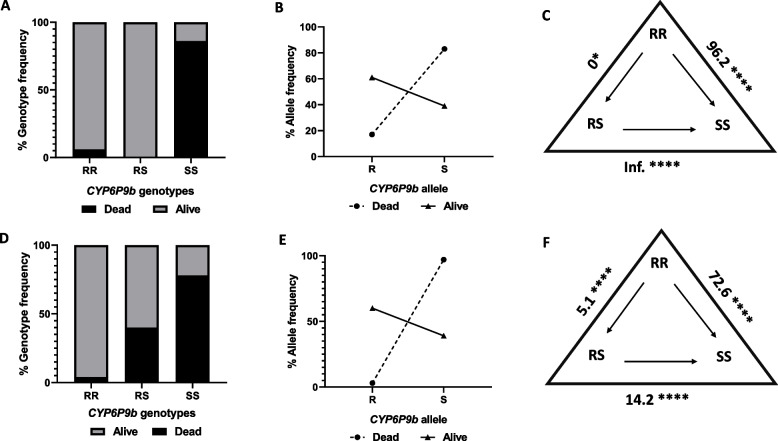
Genotype and allele frequences of *CYP6P9b* in WHO cone assays. Distribution of the *CYP6P9b* genotypes between the dead and alive FUMOZ-R/FANG F_4_ hybrids exposed to **A**) PermaNet 2.0. **D**) Sherlock. Allele frequencies of *CYP6P9b* between the dead and alive FUMOZ-R/FANG F_4_ hybrids exposed to **B**) PermaNet 2.0 and **E**) Sherlock. Estimation of odds ratio (OR) and associated significance between different genotypes and the ability to survive exposure to **C**) PermaNet 2.0 and **F**) Sherlock net. The level of significance is indicated as *P < 0.05, **P < 0.01, ***P < 0.001 as estimated by Fisher’s exact test, the arrows in the triangle indicate the direction of OR estimation and ‘Inf.’ Stands for infinity

A similar pattern was observed for the *CYP6P9b* pyrethroid-resistance marker in the dead and live FUMOZ-R/FANG F_4_ hybrid samples exposed to PermaNet 2.0. There was a strong significant association between this marker and survival. The genotypes were significantly distributed between the dead and alive groups (*X*^*2*^ = 216.8, *p* < 0.0001). Hybrids with the RR genotype for *CYP6P9b* had an increased chance to survive PermaNet 2.0 exposure compared to those with the SS genotype (OR = 96.2; CI = 33.96–256.2; *p* < 0.0001) (Table 2, Fig. 5 A and C). There was a weaker, yet significant increased survival ability of the RR genotype compared to the RS genotype (OR = 0; CI = 0–0.7; *p* = 0.0289). Overall, the R allele was strongly associated with resistance, exhibiting better odds of survival than the S allele (OR = 50.57; CI = 15.96–158.5; *p* < 0.0001) (Table 2, Fig. 5 B).

For the hybrids exposed to Sherlock bed net, there was a significant distribution of genotypes between the dead and alive groups (*X*^*2*^ = 113.5, *p* < 0.0001). Hybrids with the RR genotype were more likely to survive than those with the RS (OR = 14.2; CI = 3.4–62.3; *p* < 0.0001) and SS genotypes (OR = 5.1; CI = 2.5–9.9; *p* < 0.0001) (Table 2, Figs. 5D and F). In general, the allelic frequencies of the *CYP6P9b* resistance marker were strongly correlated with the R allele than with the S allele in terms of survival following Sherlock net exposure (OR = 7.1; CI = 3.7–13.8; *p* < 0.0001) (Table 2, Fig. 5E).

#### The combined effect of the three markers on the reduced efficacy of PermaNet 2.0 and to Sherlock net

**Fig. 6:**
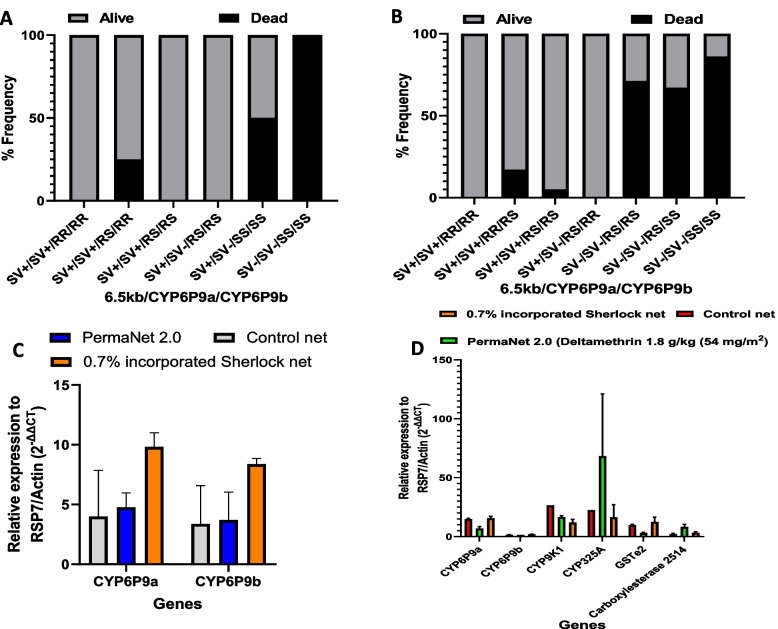
WHO Cone bioassay genotyping results showing association of the combined three genotypes of the 6.5 kb SV, CYP6P9a_R and CYP6P9b_R alleles with the resistance phenotype of FUMOZ-R/FANG F_4_ hybrids upon exposure to **A**) PermaNet 2.0, and **B**) 0.7% incorporated Sherlock net. QRT-PCR analyses of metabolic genes in **C**) FUMOZ-R/FANG F_4_ hybrids and **D**) Mibellon field-collected strains, alive after exposure to bed nets

Combining the three markers presented an additive advantage against the bio-efficacy of the PermaNet 2.0 and the Sherlock bed nets (Fig. 6 A and B, respectively). Remarkably, mosquitoes possessing triple homozygous alleles (SV + /SV + /RR/RR) for the three markers were all alive after exposure to both nets (100%), and had a greater chance of surviving exposure to PermaNet 2.0 and Sherlock bed net than those without any resistant allele (SV-/SV-/SS/SS) (PermaNet 2.0 (OR = Inf.; CI = 1980-Inf.; *p* < 0.0001), Sherlock (OR = Inf.; CI = 156.1-Inf.; *p* < 0.0001)) (Table of significance in Sup. 2b). This suggests an added advantage to individual mosquitoes possessing the three markers against the bio-efficacy of PermaNet 2.0 and Sherlock nets. Overall, the combined analysis of the three markers revealed that survival against PermaNet 2.0 exposure requires a minimum of three resistant alleles, while survival against the Sherlock bed net requires at least four resistant alleles (Fig. 6A and B, Table of significance in Sup.2c).

#### WHO tunnel tests

To evaluate the impact of *CYP* gene-mediated resistance on mortality and blood-feeding ability in host-seeking FUMOZ-R/FANG F_4_ hybrids, resistance markers were genotyped in both dead and surviving mosquitoes. To minimize bias from blood-feeding stress, only unfed mosquitoes were analysed for association between resistance markers and survival following insecticide exposure.

#### The role of 6.5 kb SV in the survival and blood-feeding ability of hybrids exposed to PermaNet 2.0 and Sherlock net

There was a significant distribution of genotypes between the dead and alive FUMOZ-R/FANG F_4_ hybrids (PermaNet 2.0 (*X*^*2*^ = 46.69, *p* < 0.0001), and Sherlock net (*X*^*2*^ = 134.5, *p* < 0.0001)). A comparative analysis of the 6.5 kb SV distribution between the dead and alive groups exposed to both nets revealed a strong association between this SV and the resistance phenotype. (Figures in Sup. 3a and Sup 3b). There was no significant difference in survival ability between SV^+^/SV^+^ hybrids and SV^+^/SV^−^ hybrids exposed to PermaNet 2.0 (OR = 1.23; CI = 0.64–2.3; *p* = 0.619). However, hybrids with either of these genotypes had a greater survival chance for PermaNet 2.0 exposure than SV^−^/SV^−^ hybrids (OR = 13.4; CI = 4.7–33.8; *p* < 0.0001 and OR = 10.9; CI = 4.0–27.1; *p* < 0.0001, for SV^+^/SV^+^ and SV^+^/SV^−^, respectively) (Table 3). However, hybrids with the SV^+^/SV^+^ genotype were more likely to survive Sherlock net exposure than those with the SV^+^/SV^−^ and SV^−^/SV^−^ genotypes (OR = 6.7; CI = 3.2–13.4; *p* < 0.0001, and, OR = Inf.; CI = 34.7-Inf., *p* < 0.0001 for SV^+^/SV^−^ and SV^−^/SV^−^ respectively). Hybrids with the SV^+^/SV^−^ genotype also exhibited greater survival ability against Sherlock net exposure than did those with the SV^−^/SV^−^ genotype (OR = Inf.; CI = 5.8-Inf.; *p* < 0.0001). Overall, individuals having the SV^+^ allele showed an increased chance of surviving exposure compared with the SV^−^ allele (PermaNet 2.0 (OR = 3; CI = 1.6–5.5; *p* = 0.0003), and Sherlock net (OR = 6.6; CI = 3.4–12.6; *p* < 0.0001)) (Table 3).

**Table 3:** Correlation between genotypes of the 6.5 kb SV, *CYP6P9a* and *CYP6P9b* and the ability of FUMOZ-R/FANG F_4_ hybrids to survive exposure to 0.7% incorporated Sherlock net and PermaNet 2.0 (WHO tunnel bioassays) ('Inf.’ Stands for ‘Infinite values’)

		**OR**	**CI P value**		**OR**	**CI P value**		**OR**	**CI**	**P value**
			***6.5 kb* SV**			***CYP6P9a***			***CYP6P9b***	
**0.7% incorporated Sherlock net**	**RR vs SS**	Inf	34.72-Inf	< 0.0001	Inf	6.28-Inf	< 0.0001	Inf	3.97-Inf	0.0002
	**RS vs SS**	Inf	5.83-Inf	< 0.0001	9.7	3.19-Inf	0.0003	Inf	3.08-Inf	0.001
	**RR vs RS**	6.73	3.23–13.42	< 0.0001	1.7	0.93–3.02	0.1037	1.3	0.41–4.19	0.3843
	**R vs S**	6.56	3.9–12.64	< 0.0001	6.56	3.39–12.64	< 0.0001	1.73	0.92–3.21	0.0892
**PermaNet 2.0**	**RR vs SS**	13.4	4.69–33.81	< 0.0001	1.5	0.53–4.51	0.5881	2.88	1.22–6.53	0.0186
	**RS vs SS**	10.9	4.04–27.07	< 0.0001	0.93	0.44–1.96	> 0.9999	3.5	1.77–7.3	0.0004
	**RR vs RS**	1.23	0.64–2.3	0.6187	1.6	0.68–3.84	0.3857	0.81	0.4–1.69	0.7054
	**R vs S**	2.99	1.64–5.47	0.0003	1.11	0.63–1.95	0.7773	1.63	0.92–2.81	0.1168

An additional analysis was conducted to assess the correlation between the 6.5 kb SV and the blood-feeding success of mosquitoes. This approach involved comparing the distribution of the SV genotypes between blood-fed and unfed groups (Figures in sup. 3a and 3b). Mosquitoes homozygous for the insertion (SV^+^/SV^+^) had a significantly greater probability of successful blood-feed than did heterozygous (SV^+^/SV^−^) individuals when exposed to PermaNet 2.0 (OR = 2.1; CI = 1.1–3.97; *p* = 0.0261) and Sherlock net (OR = 2.8; CI = 1.5–5.1; *p* = 0.0014). Similarly, SV^+^/SV^+^ mosquitoes were significantly more likely to blood-feed than SV^−^/SV^−^ individuals (PermaNet 2.0 (OR = 4.5; CI = 1.9–10.5; *p* = 0.0006); Sherlock net (OR = Inf.; CI = 7.9-Inf.; *p* < 0.0001)). No significant difference was observed between the SV^+^/SV^−^ and SV^−^/SV^−^ genotypes in their ability to traverse PermaNet 2.0 and successfully blood-feed (OR = 2.2; CI = 0.9–5.2; *p* = 0.0974). However, SV^+^/SV^−^ mosquitoes exhibited a significantly greater ability to penetrate the Sherlock net and blood-feed than SV^−^/SV^−^ mosquitoes (OR = Inf.; CI = 2.7-Inf.; *p* = 0.001). Overall, the presence of the SV^+^ allele was significantly associated with an increased odds of crossing both bed nets and successful blood-feeding compared with the SV^−^ allele (PermaNet 2.0 (OR = 2.3; CI = 1.3–4.1; *p* = 0.0078); Sherlock net (OR = 3.8; CI = 1.9–7.4; *p* = 0.0001)).

#### The role of CYP6P9a in the survival and blood-feeding ability of hybrids exposed to PermaNet 2.0 and Sherlock net

There was no significant distribution of genotypes between dead and alive mosquitoes exposed to PermaNet 2.0 (*X*^*2*^ = 3.316, *p* = 0.1905). However, mosquitoes with the RR genotype occupied the largest proportion in the alive group (60%) compared with those with the RS (48%) and SS (50%) genotypes (Figure in Sup. 3c). In contrast, there was a significant distribution of genotypes between the dead and alive mosquito groups exposed to Sherlock net (*X*^*2*^ = 134.5, *p* < 0.0001). Hybrids with the RR genotype for this marker had a greater chance to survive Sherlock net exposure than those with the SS genotype (OR = Inf.; CI = 6.3-Inf.; *p* < 0.0001) (figure in Sup. 3d). Similarly, the RS genotype had a greater chance of surviving exposure than the SS genotype (OR = Inf.; CI = 3.2- Inf.; *p* = 0.0003) suggesting that one R-allele is sufficient to confer cross-resistance to Sherlock. Compared with the S-allele, the R-allele for *CYP6P9a* generally had a greater chance of surviving exposure to Sherlock net (OR = 6.6; CI = 3.4–12.6; *p* < 0.0001) (Table 3).

There was no significant association between this marker and the ability of hybrids to successfully blood-feed. However, a genotype–phenotype association was established by evaluating the survival of blood-fed mosquitoes post exposure. This analysis compared the proportion of FUMOZ-R/FANG F_4_ hybrids that survived after blood-feeding to those that died after feeding (Figures in Sup. 3 g and table of significance in Sup.3 h). Hybrids with the RR genotype were significantly more likely to survive post blood-feeding than were those with the RS genotype when exposed to PermaNet 2.0 (OR = 10.62; CI = 4.7–22.5; *p* < 0.0001) and Sherlock net (OR = 3.9; CI = 2.1–7.1; *p* < 0.0001). Similarly, RR hybrids presented a pronounced survival advantage over SS individuals (PermaNet 2.0 (OR = 46; CI 18.5–105.0; *p* < 0.0001) and Sherlock net (OR = 19.5; CI = 9.3–38.5; *p* < 0.0001). This finding suggests that possessing two resistant alleles provides an added survival advantage post blood-feeding. Additionally, the RS hybrids had a significantly greater chance of survival than the SS individuals (PermaNet 2.0 (OR = 4.3, CI 2.3 to 7.9, *p* < 0.0001); Sherlock net (OR = 5.03; CI = 2.55–10.12; *p* = 0.0177)) suggesting that possessing one R-allele could significantly reduce the efficacy of these bed nets by increasing the chance of successful penetration, blood-feeding, and survival post -feeding.

#### The role of CYP6P9b in the survival and blood-feeding ability of hybrids exposed to PermaNet 2.0 and Sherlock net

There was a significant distribution of the different genotypes in the dead and alive groups (PermaNet 2.0 (*X*^*2*^ = 16.9, p = 0.0002); Sherlock net (*X*^*2*^ = 78.74, *p* < 0.0001)). The hybrids possessing the RR genotype were more likely to survive exposure to PermaNet 2.0 than those with the SS genotype (PermaNet 2.0 (OR = 2.9; CI = 1.2–6.5; *p* = 0.0186); Sherlock net (OR = Inf.; CI = 3.97-Inf., *p* = 0.0002)). Similarly, hybrids with the RS genotype were more likely to survive exposure to bed nets than those with the SS genotype (PermaNet 2.0 (OR = 3.5; CI = 1.8–7.3; *p* = 0.0004); Sherlock net (OR = Inf.; CI = 3.08-Inf.; *p* = 0.001)) (Figures in Sup 3e and 3f).

A similar trend was observed for the *CYP6P9b* (figures in Sup. 3e and Sup. 3f) when assessing the effects of this marker on the ability of mosquitoes to survive insecticide exposure post-blood-feeding. Compared with hybrids with the SS genotype, those with the RR genotype were more likely to survive after successfully penetrating the bed net barrier and blood-feeding on bait (PermaNet 2.0 (OR = 3.5; CI = 1.9–6.2; *p* < 0.0001); Sherlock net (OR = 29.33; CI = 13.6–61.7; *p* < 0.0001)). Additionally, hybrids with the RR genotype had a more significant association than those with the RS genotype (OR = 13.04; CI = 6.3–26.6, *p* < 0.0001). Similarly, the RS genotype had a greater chance of successfully penetrating, blood feeding and surviving than did the SS genotype (OR = 3.96, CI = 2.2 to 6.9, p < 0.0001) (Table of significance in Sup. 3 h). This finding suggests that having two R-alleles for this gene provides an added advantage for penetration, blood-feeding, and survival after blood-feeding.

#### The combined effect of the three markers on the survival and blood-feeding ability of hybrids exposed to PermaNet 2.0 and Sherlock net

**Fig. 7:**
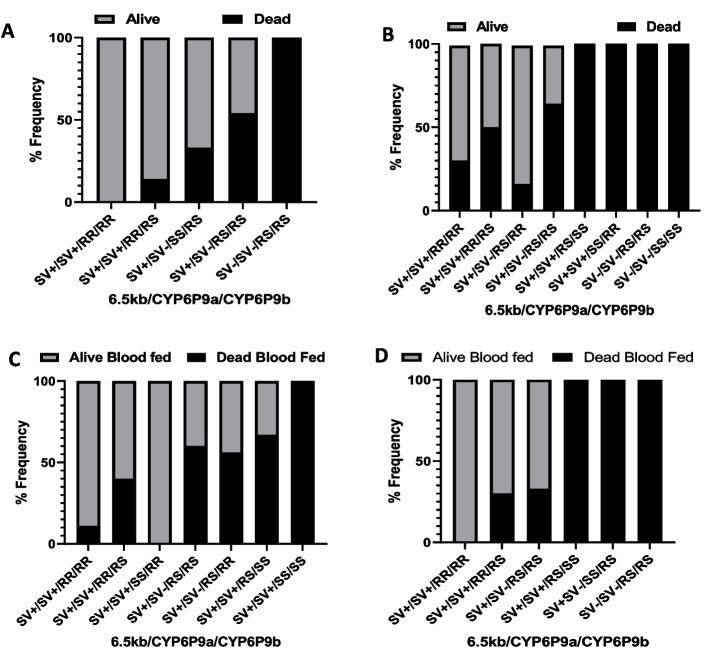
WHO tunnel bioassay genotyping results showing association of the three markers combined with resistance. Genotype frequencies of the combined genotypes 6.5 kb SV, CYP6P9a_R and CYP6P9b_R alleles among FUMOZ-R/FANG F_4_ hybrids exposed to **A**) Permanent 2.0, and **B**) 0.7% incorporated Sherlock net. Association of the combined three markers among FUMOZ-R/FANG F_4_ hybrids and ability to survive after successfully penetrating through bed net and blood-feeding **C**) 0.7% incorporated Sherlock net, and **D**) PermaNet 2.0

As with the other bioassays, combining the three markers resulted in an increased survival advantage against both PermaNet 2.0 and the Sherlock bed net in WHO tunnel tests (Figs. 7A and 7B, respectively). Overall, mosquitoes triple homozygous with 6.5 kb SV, *CYP6P9a,* and *CYP6P9b* (SV^+^/SV^+^/RR/RR) had the greatest chance of surviving exposure to the different bed nets (Table of significance Sup. 3i). This result suggests that there is an additive advantage in the resistance to pyrethroids and cross-resistance to Sherlock when the six resistant alleles are present.

Like the individual markers, the combination of the three resistance markers showed no clear correlation with blood-feeding ability. However, a high proportion of hybrids possessing triple homozygous resistant alleles successfully penetrated the net barrier, fed on the bait, and survived, with 89% survival following Sherlock net exposure and 100% survival with PermaNet 2.0 exposure (Fig. 7C and 7D). FUMOZ-R/FANG F_4_ hybrids triple homozygous with 6.5 kb SV, *CYP6P9a,* and *CYP6P9b* (SV^+^/SV^+^/RR/RR) had the highest chance of surviving post-exposure and blood feeding (Table of significance in Sup. 3j).

#### Relative expressions of* CYP6P9a *and* CYP6P9b *and reduced bio-efficacy of pyrethroid and Sherlock insecticides via* qRT-PCR*

qRT-PCR was performed to assess the role of the upregulation of metabolic genes in the resistance of field strains and hybrids exposed to insecticide-impregnated bottles and bed nets. The expression profiles of the known genes associated with pyrethroid metabolism *CYP325A*, *CYP9K1*, *GSTe2*, Carboxylesterase 2514, *CYP6P9a*, and *CYP6P9b* were analysed in field strains, whereas the expression of *CYP6P9a* and *CYP6P9b* was evaluated in the F_4_ hybrid samples that were alive after exposure to the different insecticide bottles and bed nets.

For the bottle assays, qRT-PCR analysis revealed resistance-associated genes to be upregulated relative to the FANG (susceptible) strain and the negative control groups. The genes upregulated when the field samples were exposed to permethrin treated bottles were; *CYP6P9a* (FC = 28.3 ± 10.3), *CYP6P9b* (FC = 22.2 ± 3.6), *CYP325A* (FC = 20.3 ± 23.7), and *CYP9K1* (FC = 11.3 ± 2.1) (Fig. 2D). Compared with the susceptible (FANG) and negative controls, the F_4_ hybrids presented a significant upregulation of *CYP6P9a* and *CYP6P9b* (Fig. 2E). Survival of exposure to the 5xDC Sherlock showed the highest fold changes for *CYP6P9a* (FC = 45.6 ± 4.5; t = 12.97; *p* = 0.001; df = 3) and *CYP6P9b* (FC = 91.7 ± 33.4; t = 4.25; *p* = 0.0238; df = 3) compared to the negative control group (*CYP6P9a* (FC = 12.1 ± 1.5) and *CYP6P9b* (FC = 16.5 ± 1.5)). The 1xDC Sherlock exposed equally showed a significant upregulation of *CYP6P9a* (FC = 25 ± 1.8; t = 8.87; *p* = 0.003; df = 3) and *CYP6P9b* (FC = 44 ± 0.2; t = 24.04; *p* = 0.0002; df = 3) relative to the negative control. These genes were also significantly upregulated in the alive hybrids exposed to permethrin-impregnated bottles (*CYP6P9a* (FC = 35.4 ± 4.1; t = 9.5; *p* = 0.0025, df = 3), and, *CYP6P9b* (FC = 61.6 ± 3.6; t = 20.4; *p* = 0.0003, df = 3)) compared to the negative control group (*CYP6P9a*; FC = 35.4 ± 4.1 and *CYP6P9b*; FC = 61.6 ± 3.6).

For the alive PermaNet 2.0 net exposed field strains, only two genes presented a higher fold than the untreated net: *CYP325A* (FC = 68 ± 74.7) and carboxylesterase 2514 (FC = 8.2 ± 3.7) (Fig. 2D). For the surviving Sherlock net-exposed field strains, *CYP6P9a* was the most upregulated gene (FC = 15.7 ± 2.6). However, no gene presented a significant increase in expression compared to that of the untreated net. qRT-PCR analysis of the alive hybrid strains exposed to bed nets revealed an increased expression of resistance genes, compared with those exposed to the untreated net, although the differences were not statistically significant (*p* > 0.05). The highest fold changes (FC) were observed in alive hybrids exposed to Sherlock net (9.8 ± 1.2 (*CYP6P9a*) and 8.4 ± 0.5 (*CYP6P9b*)) (Fig. 3C). These results suggest that there is no significant insecticide-induced expression of resistance genes in response to bed net exposure.

## Discussion

In recent years, the fight against malaria via vector control has helped reduce the malaria burden with pyrethroid insecticides being at the top of the list of insecticides recommended in public health due to their safety. However, the emergence and spread of pyrethroid-resistant mosquitoes remains a huge threat to the efficacy of currently implemented pyrethroid-based vector control tools [39, 40]. This has resulted in setbacks in the gains previously made by malaria control programs toward malaria elimination. The objective of integrated resistance management (IRM) via the development of novel insecticide formulations has been strongly encouraged by the Global Plan for Insecticide Resistance Management. Since 2005, the IVCC with its main industry partners and funders has been working hard towards establishing a rich portfolio of novel insecticides and vector control products. This has included an exploration of the potential of the novel mitochondrial complex I inhibitor insecticide Sherlock, as a possible active ingredient for sustained vector control and a move towards malaria eradication [17, 18]. One of the biggest challenges to these efforts, for both repurposed and novel insecticides, is the phenomenon of insecticide ‘cross-resistance’ [41]. Overuse of pyrethroids has led to the evolution and spread of resistance mechanisms that act not only to reduce the bio-efficacy of pyrethroids but also may reduce the bio-efficacy of other insecticides from different classes with unrelated modes of action [10, 41]. Therefore, before devoting significant resources to the development of a non-pyrethroid insecticide for vector control, it is essential to ensure that the target mosquito populations are not already cross-resistant and, if cross-resistance exists, to evaluate the impact that this would have on the bio-efficacy of any products into which they might be formulated. In this study, we first comparatively assessed the efficacy of the novel insecticide Sherlock compared to that of a pyrethroid insecticide via CDC bottle bioassays to detect cross-resistance. We then evaluated the efficacy of ITNs impregnated with the Sherlock insecticide. We investigated the molecular mechanisms involved in cross-resistance to Sherlock, focusing on known pyrethroid-resistance markers; 6.5 kb SV, *CYP6P9a*, *CYP6P9b*, and GSTe2. Finally, we assessed whether the upregulation of known detoxification genes in *An. funestus* s.s. mosquitoes contribute to resistance to pyrethroids and cross-resistance to Sherlock.

### Laboratory An. funestus s.s. strains exhibit resistance to pyrethroids, and Sherlock contrasting to field-collected An. gambiae s.s. strains

Owing to the resistance phenotype of the *An. funestus* s.s. strains, there was an overall low bio-efficacy of the Sherlock insecticide, in bioassays due to cross-resistance. This was in contrast to our observations with the *An. gambiae* s.s. from Nkolondom following exposure to Sherlock, even though this population has been shown to be highly resistant to pyrethroids [29, 42]; this resistance was confirmed in the pyrethroid bioassay results obtained from this study.

The high-intensity cross-resistance observed in the pyrethroid-resistant *An. funestus* s.s. FUMOZ-R strain against Sherlock suggests the presence of strong resistance mechanisms. The reduced efficacy at elevated diagnostic concentrations may reflect a combination of target saturation and insecticide-induced stress responses that activate alternative survival pathways in mosquitoes. This observation points to the need for deeper molecular investigations. Transcriptomic approaches, such as RNA sequencing, could further identify these pathways, potentially identifying upregulated genes beyond known detoxification gene families. Other protein families, such as oxidative stress, cuticular, digestive, and heat shock proteins, have been upregulated to pyrethroid insecticide resistance [22]. The poor performance of PermaNet 2.0, PermaNet 3.0 (side), and Sherlock nets in WHO cone bioassays highlights resistance in both laboratory hybrid strains and even higher in field-collected *An. funestus* populations. The higher resistance/cross-resistance in field-collected strains may reflect the existence of multiple resistance mechanisms. A full recovery in susceptibility was observed when all strains were exposed to PermaNet 3.0 roof netting which includes the synergist PBO, further implicating the role of *CYP*-mediated resistance and reinforcing the value of synergist-based nets in vector control. These results are consistent with previous studies evaluating the efficacy of these pyrethroid/PBO-treated ITNs on *An. funestus* FUMOZ-R/FANG hybrids with a similar genetic background [24–26], as well as with Mibellon field strains [43]. The significant role of *CYP* genes in insecticide resistance has also been demonstrated in *An. funestus* mosquito populations from Uganda [5], Ghana [6], and Malawi [7]. This further highlights the gross impact of *CYP* gene-mediated resistance in *An. funestus* s.s. against pyrethroids. WHO tunnel tests generate bio-efficacy data that are typically more predictive of the bio-efficacy of bed nets under field conditions. These WHO tunnel tests revealed higher mortality rates in hybrids compared to WHO cone assays, likely due to the prolonged insecticide exposure and repeated contact with ITNs during host-seeking behaviour. The Sherlock bed net performed particularly well, not only increasing mortality but also substantially inhibiting blood feeding of hybrids. These findings suggest that, despite the partial resistance observed following exposure to the Sherlock bed net, it could still offer significant personal protection against mosquito bites. The high blood feeding inhibition highlights the potential for the Sherlock net having a novel chemistry, to reduce malaria transmission even in the presence of moderate resistance. As previously reported, the PBO-containing roof panel of the PermaNet 3.0 net demonstrated complete blood feeding inhibition and full mortality, reiterating the utility of synergists-incorporated nets to manage metabolic resistance [24, 26].

### Pyrethroid resistance-associated *CYP* genes mediate the reduced bio-efficacy of pyrethroid and Sherlock net formulations

To understand the observed phenotypic resistance of FUMOZ-R/FANG F_4_ hybrids, the previously described southern African-originating pyrethroid-resistance markers in the intergenic region of *CYP6P9a* and *CYP6P9b* [24, 25] were comparatively genotyped between dead and alive bioassay-generated samples. CDC bottle bioassays and WHO cone bioassays revealed a strong association between these markers and the ability to survive pyrethroid and Sherlock bed net exposure. To further demonstrate the role of these markers in pyrethroid and Sherlock efficacy, WHO tunnel tests revealed that *An. funestus* s.s. with resistant alleles survived exposure after blood feeding. This would not only lead to an increase in malaria transmission by resistant mosquitoes but also, they could either live long enough to successfully produce more progeny or transmit malaria by biting other non-infected individuals. These results are similar to those previously obtained for 6.5 kb SV [26], *CYP6P9a* [24], and *CYP6P9b* [25], while performing experimental hut trials with the deltamethrin-treated bed net; PermaNet 2.0. This finding further highlights how such resistance markers could lead to a stagnation to the gains that have been made in reducing malaria transmission. Studies have linked these resistance markers in FANG/FUMOZ hybrids to an increased chance of blood-feeding [44] even in the presence of pyrethroid-impregnated bed nets [24–26]. We also found that these markers are more strongly associated with pyrethroid resistance at shorter exposure times. In WHO cone bioassays with a short 3-min exposure time, we observed that the 6.5 kb insertion was more associated with PermaNet 2.0 reduced efficacy than with Sherlock. This insertion, located in the intergenic region of *CYP6P9a* and *CYP6P9b*, has been shown to significantly enhance the upregulation of these detoxification enzymes, with studies reporting up to a 5.2-folds (*p* = 0.005) and 4-folds (*p* = 0.001) increase in *CYP6P9a* and *CYP6P9b* expression levels comparing homozygotes with the SV to homozygotes without the SV [26]. These enzymes may have a higher affinity for pyrethroids such as deltamethrin (the active ingredient in PermaNet 2.0) and can break them down more efficiently than Sherlock, resulting in higher survival rates when mosquitoes are exposed to PermaNet 2.0 than when they are exposed to the Sherlock net [9, 28]. However, with prolonged exposure in tunnel tests, these markers were strongly associated with a reduced efficacy of the Sherlock net. This difference may be attributed to the different modes of action of Sherlock and pyrethroid insecticides. While pyrethroids disrupt the voltage-gated sodium channels of insect nerve cells [45], Complex I inhibitors disrupt ATP synthesis by inhibiting NADH:ubiquione oxidoreductase activity, a critical step in the mitochondrial electron transport chain [19]. This inhibition disrupts electron transfer to downstream complexes, preventing the establishment of a proton gradient essential for H + exchange and ATP production. This process may exhibit a rather slower onset of action than the rapid-acting pyrethroid insecticides which cause immediate neuronal hyperexcitation and insect paralysis. Additionally, the *CYP* genes may respond differently to the two insecticides, mechanistically. The molecular basis of *CYP*-gene mediated resistance may involve an increase in the copy number of metabolic enzymes, which can either sequester or rapidly metabolize various classes of insecticides [41]. Furthermore, in the context of cross-resistance, other studies have demonstrated that these CYPs confer cross-resistance to carbamate insecticides that normally target acetylcholinesterase in mosquito neuronal cells [9, 10]. This further highlights the importance of assessing the risk of pyrethroid-resistance markers offering cross-resistance to other insecticide classes currently used or used in vector control tools. The impact of these markers in the context of Sherlock insecticide exposure could be further assessed in terms of fitness cost parameters, such as longevity, as has been demonstrated for pyrethroid-impregnated bed nets [46–48].

### The L119F_*GSTe2* DDT/pyrethroid-resistance marker mediates resistance against pyrethroids but not against Sherlock

Genotyping of the L119F_*GSTe2* mutation in the cone bioassay samples generated from exposed field samples against PermaNet 2.0 revealed a clear correlation between this marker and pyrethroid resistance. The RR genotype had the greatest chance of surviving exposure than the RS. However, there was no association between this marker and Sherlock resistance, even though all the mosquitoes with the RR genotype were found in the alive group. Previous studies have also linked this marker to DDT resistance [28, 37]. This means that this marker could be used to evaluate the performance of other insecticide-based vector control tools. We found no association between this resistance maker and cross-resistance to the Sherlock insecticide.

### The 6.5 kb SV, *CYP6P9a* and *CYP6P9b* combine to exacerbate resistance to pyrethroids and Sherlock insecticide

Evaluating the combined effect of these resistance markers on the efficacy of pyrethroids and Sherlock insecticide clearly revealed that they collectively contribute to a significant degree of cross-resistance, as revealed by the genotyping results from the three bioassays. This could be partly because of the 6.5 kb SV located between *CYP6P9a* and *CYP6P9b*, which acts as an enhancer [26]. Previous reports have not only highlighted the importance of enhancers on neighbouring genes [20, 49] but also showed that this insertion contains cis-regulatory elements with transcription factor binding sites [26] such as CnCC/Maf [50], GC and CCAAT [24, 26]. Hence, the 6.5 kb SV regulates the over-expression of the tandemly duplicated *CYP* genes *CYP6P9a* and *CYP6P9b*, which are key pyrethroid resistance genes [24, 25, 28, 51, 52]. Regardless of the enhancer effect of the 6.5 kb SV, the overexpression role of these *CYP* genes in pyrethroid resistance has already been well established via heterologous expression in *Escherichia coli* [28] and molecular docking simulations [27], indicating that these genes directly metabolize pyrethroids. Evaluating the combined effect of these markers on the ability to survive after blood feeding revealed that hybrids with the triple homozygous resistance genotype for the three markers had the greatest chance of surviving after exposure to the deltamethrin- and sherlock-impregnated bed nets. This reflects a key challenge in vector control programs, hindering progress in malaria elimination, and aligns with previous reports from Bioko Island linking the upregulation of the pyrethroid-resistance gene *CYP9K1* to reduced control effectiveness [53]. To avert the reduced efficacy of pyrethroid and Sherlock bed nets caused by *CYP* gene-mediated resistance, co-deploying with piperonyl butoxide, a *CYP* gene inhibitor, could be a potential solution.

### The upregulation of *CYP* genes contributes to the reduced efficacy of pyrethroid and the Sherlock net formulations

The qRT-PCR results from mosquitoes exposed in the CDC bottle bioassay with the F_4_ hybrids exposure clearly revealed that the overexpression of *CYP6P9a* and *CYP6P9b* is linked to pyrethroid resistance and Sherlock cross-resistance. Previous studies have shown that the upregulation of these metabolic genes in *An. funestus* strongly correlates to pyrethroid resistance [9, 24–26, 28]. The increased expression observed in the hybrids exposed to insecticide bottles compared with the negative control suggests that these genes are induced in response to pyrethroid, and Sherlock insecticide and that the intensity of this induction increases with increased insecticide concentration. While previous studies have reported a correlation between the inducibility of these CYPs and their role in pyrethroid detoxification [9, 27, 28], *in vitro* metabolic assays could provide direct evidence confirming their involvement in detoxifying the Sherlock insecticide. The short duration of insecticide exposure with WHO cone assays limits the ability to detect the link between these genes' overexpression and the ITNs' bio-efficacy. Similarly, these markers have been reported to be overexpressed in pyrethroid-resistant *An. funestus* s.s. mosquitoes from southern Africa against pyrethroids [40, 54]. A recent report also revealed that the *CYP6P9a* marker is overexpressed in response to carbamates [10]. The link between increased overexpression of known detoxification genes and high pyrethroid resistance was investigated in field strains with no differences observed between 1xDC and 5 × DC. The *CYP* gene; *CYP325A* which was previously shown to be involved in pyrethroid resistance among central African *An. funestus* populations [55], was constitutively expressed in field strains exposed to permethrin and Sherlock bottles. Further transcriptomics analyses can be performed with the mosquitoes resistant to sherlock to confirm the upregulation of *CYP6P9a/b* genes against sherlock, identify other candidates involved in Sherlock resistance, as well as functionally validate via *in vivo* heterologous expression in *Drosophila melanogaster* to establish the exact role of detoxification genes in resistance.

## Conclusion

This study explored the association between established resistance markers found in pyrethroid-resistant *An. funestus* s.s. and cross-resistance to a novel mitochondrial Complex I inhibitor insecticide, Sherlock. Standard susceptibility assays were used to establish that pyrethroid-resistant *An. funestus* s.s. and *An. gambiae* s.s. field populations in Cameroon, in which these resistance markers are absent, are susceptible to this insecticide, whilst the pyrethroid-resistant *An. funestus* s.s. FUMOZ-R strain, which possesses these resistance markers, exhibited cross-resistance. The Sherlock insecticide may, however, still be of use in localities where these resistance markers are absent. Further molecular investigations to delimit the molecular basis of cross-resistance revealed that both the presence and overexpression of pyrethroid resistance markers *CYP6P9a/b* are clearly associated with survival following exposure to the Sherlock insecticide. These markers were also shown to be linked to the reduced efficacy of the prototype Sherlock net formulation. Hence, maximising the utility of an incorporated Sherlock ITN formulation may require co-formulation with the synergist PBO.

## Supplementary Information

Supplementary Material 1

Supplementary Material 2

Supplementary Material 3

Supplementary Material 4

Supplementary Material 5

Supplementary Material 6

Supplementary Material 7

Supplementary Material 8

## Data Availability

The datasets supporting the conclusions of this article are included within the published article (and its additional files).

## Abbreviations

DC: Discriminating Concentration
PBO: Piperonyl Butoxide
IRS: Indoor Residual Spray
ITN: Insecticide-Treated Net
IVCC: Innovative Vector Control Consortium
SV: Structural Variant
FUMOZ-R: Pyrethroid-resistant *An. funesutus* colonised from Mozambique
FANG-S: Insecticide susceptible *An. funestus* colonised from Angola
RR: Homozygous resistant
RS: Heterozygous resistant
SS: Homozygous susceptible
OR: Odds ratio

## References

[1] WHO, World malaria report. 2024, Geneva: World Health Organization.

[2] Bhatt S, et al. The effect of malaria control on *Plasmodium falciparum* in Africa between 2000 and 2015. Nature. 2015;526(7572):207–11. 26375008 10.1038/nature15535 PMC4820050

[3] Hemingway J. Resistance: a problem without an easy solution. Pestic Biochem Physiol. 2018;151:73–5. 30704716 10.1016/j.pestbp.2018.08.007

[4] Protopopoff N, et al. Effectiveness of a long-lasting piperonyl butoxide-treated insecticidal net and indoor residual spray interventions, separately and together, against malaria transmitted by pyrethroid-resistant mosquitoes: a cluster, randomised controlled, two-by-two factorial design trial. Lancet. 2018;391(10130):1577–88. 29655496 10.1016/S0140-6736(18)30427-6 PMC5910376

[5] Tchouakui M, et al. Pyrethroid resistance aggravation in Ugandan malaria vectors is reducing bednet efficacy. Pathogens. 2021;10(4):415. 33915866 10.3390/pathogens10040415 PMC8065452

[6] Mugenzi LMJ, et al. Escalating pyrethroid resistance in two major malaria vectors *Anopheles funestus* and *Anopheles gambiae* (s.l.) in Atatam, Southern Ghana. BMC Infect Dis. 2022;22(1):799. 36284278 10.1186/s12879-022-07795-4 PMC9597992

[7] Menze BD, et al. Marked aggravation of pyrethroid resistance in major malaria vectors in Malawi between 2014 and 2021 is partly linked with increased expression of P450 alleles. BMC Infect Dis. 2022;22(1):660. 35907831 10.1186/s12879-022-07596-9 PMC9338535

[8] Ranson H, Lissenden N. Insecticide resistance in African Anopheles mosquitoes: a worsening situation that needs urgent action to maintain malaria control. Trends Parasitol. 2016;32(3):187–96. 26826784 10.1016/j.pt.2015.11.010

[9] Ibrahim SS, et al. The P450 CYP6Z1 confers carbamate/pyrethroid cross-resistance in a major African malaria vector beside a novel carbamate-insensitive N485I acetylcholinesterase-1 mutation. Mol Ecol. 2016;25(14):3436–52. 27135886 10.1111/mec.13673 PMC4950264

[10] Mugenzi LMJ, et al. The duplicated P450s CYP6P9a/b drive carbamates and pyrethroids cross-resistance in the major African malaria vector *Anopheles funestus*. PLoS Genet. 2023;19(3): e1010678. 36972302 10.1371/journal.pgen.1010678 PMC10089315

[11] Nkemngo FN, et al. Multiple insecticide resistance and Plasmodium infection in the principal malaria vectors Anopheles funestus and Anopheles gambiae in a forested locality close to the Yaounde airport. Cameroon Wellcome Open Res. 2020;5:146. 33204845 10.12688/wellcomeopenres.15818.1 PMC7667521

[12] Menze BD, et al. Multiple insecticide resistance in the malaria vector *Anopheles funestus* from northern Cameroon is mediated by metabolic resistance alongside potential target site insensitivity mutations. PLoS One. 2016;11(10): e0163261. 27723825 10.1371/journal.pone.0163261 PMC5056689

[13] WHO, Global technical strategy for malaria control 2016–2030, 2021 update *. * Geneva: World Health Organization; 2021. Licence: CC BY-NC-SA 3.0 IGO. Report No.: 978-92-4-003135-7. p. 40. https://www.who.int/publications/i/item/9789240031357.

[14] WHO, World malaria report 2023. Geneva: World Health Organization; 2023. Licence: CC BY-NC-SA 3.0 IGO. Report No.: 9789240086173, 283. https://www.who.int/publications/i/item/9789240086173.

[15] WHO, World malaria report 2022. Geneva World Health Organization; 2022. Licence: CC BY-NC-SA 3.0 IGO Repor t No.: 978-92-4-006489-8. p. 356. https://www.who.int/publications/i/item/9789240064898.

[16] WHO, Global plan for insecticide resistance management in malaria vectors : executive summary*.* Geneva: World Health Organization ; 2012: p. 22. https://www.who.int/publications/i/item/WHO-HTM-GMP-2012.5.

[17] Hemingway J, et al. The Innovative Vector Control Consortium: improved control of mosquito-borne diseases. Trends Parasitol. 2006;22(7):308–12. 16713358 10.1016/j.pt.2006.05.003

[18] Rowland M, et al. A new long-lasting indoor residual formulation of the organophosphate insecticide pirimiphos methyl for prolonged control of pyrethroid-resistant mosquitoes: an experimental hut trial in Benin. PLoS One. 2013;8(7): e69516. 23936033 10.1371/journal.pone.0069516 PMC3720653

[19] Lees RS, et al. New insecticide screening platforms indicate that mitochondrial complex I inhibitors are susceptible to cross-resistance by mosquito P450s that metabolise pyrethroids. Sci Rep. 2020;10(1):16232. 33004954 10.1038/s41598-020-73267-x PMC7530702

[20] Nauen R, et al. The Role of Cytochrome P450s in Insect Toxicology and Resistance. Annu Rev Entomol. 2022;67:105–24. 34590892 10.1146/annurev-ento-070621-061328

[21] Scott JG. Cytochromes P450 and insecticide resistance. Insect Biochem Mol Biol. 1999;29(9):757–77. 10510498 10.1016/s0965-1748(99)00038-7

[22] Wondji CS, et al. RNAseq-based gene expression profiling of the *Anopheles funestus* pyrethroid-resistant strain FUMOZ highlights the predominant role of the duplicated CYP6P9a/b cytochrome P450s. G3 Genes|Genomes|Genetics. 2022. 10.1093/g3journal/jkab352. 34718535 10.1093/g3journal/jkab352 PMC8727960

[23] Weedall GD, et al. An Africa-wide genomic evolution of insecticide resistance in the malaria vector *Anopheles funestus* involves selective sweeps, copy number variations, gene conversion and transposons. PLoS Genet. 2020;16(6): e1008822. 32497040 10.1371/journal.pgen.1008822 PMC7297382

[24] Weedall GD, et al. A cytochrome P450 allele confers pyrethroid resistance on a major African malaria vector, reducing insecticide-treated bednet efficacy. Sci Transl Med. 2019;11(484):7386. 10.1126/scitranslmed.aat7386 30894503

[25] Mugenzi LMJ, et al. Cis-regulatory CYP6P9b P450 variants associated with loss of insecticide-treated bed net efficacy against *Anopheles funestus*. Nat Commun. 2019;10(1):4652. 31604938 10.1038/s41467-019-12686-5 PMC6789023

[26] Mugenzi LMJ, et al. A 65-kb intergenic structural variation enhances P450-mediated resistance to pyrethroids in malaria vectors lowering bed net efficacy. Mol Ecol. 2020;29(22):4395–411. 32974960 10.1111/mec.15645

[27] Ibrahim SS, et al. Allelic variation of cytochrome P450s drives resistance to bednet insecticides in a major malaria vector. PLoS Genet. 2015;11(10): e1005618. 26517127 10.1371/journal.pgen.1005618 PMC4627800

[28] Riveron JM, et al. Directionally selected cytochrome P450 alleles are driving the spread of pyrethroid resistance in the major malaria vector *Anopheles funestus*. Proc Natl Acad Sci U S A. 2013;110(1):252–7. 23248325 10.1073/pnas.1216705110 PMC3538203

[29] Tchouakui M, et al. High efficacy of chlorfenapyr-based net Interceptor((R)) G2 against pyrethroid-resistant malaria vectors from Cameroon. Infect Dis Poverty. 2023;12(1):81. 37641108 10.1186/s40249-023-01132-w PMC10463949

[30] Morgan JC, et al. Pyrethroid resistance in an *Anopheles funestus* population from Uganda. PLoS One. 2010;5(7): e11872. 20686697 10.1371/journal.pone.0011872 PMC2912372

[31] Hunt RH, et al. Laboratory selection for and characteristics of pyrethroid resistance in the malaria vector *Anopheles funestus*. Med Vet Entomol. 2005;19(3):271–5. 16134975 10.1111/j.1365-2915.2005.00574.x

[32] Wondji CS, et al. Mapping a quantitative trait locus (QTL) conferring pyrethroid resistance in the African malaria vector Anopheles funestus. BMC Genomics. 2007;8:34. 17261170 10.1186/1471-2164-8-34 PMC1790900

[33] CDC, Centers for Disease Control and Prevention. Guideline for evaluating insecticide resistance in vectors using the CDC bottle bioassay. Atlanta, GA: Centers for Disease Control and Prevention; 2012. 28 p. https://stacks.cdc.gov/view/cdc/21777.

[34] WHO, Manual for monitoring insecticide resistance in mosquito vectors and selecting appropriate interventions. Geneva: World Health Organization; 2022. Licence: CC BY-NC-SA 3.0 IGO. Report No.: 978-92-4-005108-9 . p 70. https://www.who.int/publications/i/item/9789240051089.

[35] WHO, Guidelines for laboratory and field-testing of long-lasting insecticidal nets. 2013, Geneva: World health organization. 102.

[36] Livak KJ. Organization and mapping of a sequence on the *Drosophila melanogaster* X and Y chromosomes that is transcribed during spermatogenesis. Genetics. 1984;107(4):611–34. 6430749 10.1093/genetics/107.4.611 PMC1202380

[37] Tchouakui M, et al. A marker of glutathione S-transferase-mediated resistance to insecticides is associated with higher *Plasmodium* infection in the African malaria vector *Anopheles funestus*. Sci Rep. 2019;9(1):5772. 30962458 10.1038/s41598-019-42015-1 PMC6453935

[38] Schmittgen TD, Livak KJ. Analyzing real-time PCR data by the comparative c(T) method. Nat Protoc. 2008;3(6):1101–8. 18546601 10.1038/nprot.2008.73

[39] Ibrahim SS, et al. Pyrethroid resistance in the major malaria vector Anopheles funestus is exacerbated by overexpression and overactivity of the *P450 CYP6AA1* across Africa. Genes. 2018;9(3): 140. 29498712 10.3390/genes9030140 PMC5867861

[40] Riveron JM, et al. Escalation of pyrethroid resistance in the malaria vector *Anopheles funestus* induces a loss of efficacy of piperonyl butoxide-based insecticide-treated nets in Mozambique. J Infect Dis. 2019;220(3):467–75. 30923819 10.1093/infdis/jiz139 PMC6603977

[41] Liu N. Insecticide resistance in mosquitoes: impact, mechanisms, and research directions. Annu Rev Entomol. 2015;60:537–59. 25564745 10.1146/annurev-ento-010814-020828

[42] Tene-Fossog B, et al. Temporal variation of high-level pyrethroid resistance in the major malaria vector *Anopheles gambiae* s.l. in Yaounde, Cameroon, is mediated by target-site and metabolic resistance. Med Vet Entomol. 2022;36(3):247–59. 35521949 10.1111/mve.12577 PMC9545389

[43] Menze BD, et al. Bionomics and insecticides resistance profiling of malaria vectors at a selected site for experimental hut trials in central Cameroon. Malar J. 2018;17(1):317. 30165863 10.1186/s12936-018-2467-2 PMC6117958

[44] Nouage L, et al. Influence of GST- and P450-based metabolic resistance to pyrethroids on blood feeding in the major African malaria vector Anopheles funestus. PLoS One. 2020;15(9): e0230984. 32946446 10.1371/journal.pone.0230984 PMC7500606

[45] Soderlund DM. Molecular mechanisms of pyrethroid insecticide neurotoxicity: recent advances. Arch Toxicol. 2012;86(2):165–81. 21710279 10.1007/s00204-011-0726-x PMC3218237

[46] Ngongang-Yipmo ES, et al. Reduced performance of community bednets against pyrethroid-resistant *Anopheles funestus* and *Anopheles gambiae*, major malaria vectors in Cameroon. Parasit Vectors. 2022;15(1): 230. 35754045 10.1186/s13071-022-05335-2 PMC9233849

[47] Tchakounte A, et al. Exposure to the insecticide-treated bednet PermaNet 2.0 reduces the longevity of the wild African malaria vector *Anopheles funestus* but GSTe2-resistant mosquitoes live longer. PLoS One. 2019;14(3): e0213949. 30870507 10.1371/journal.pone.0213949 PMC6417719

[48] Tchouakui M, et al. Cytochrome P450 metabolic resistance (CYP6P9a) to pyrethroids imposes a fitness cost in the major African malaria vector *Anopheles funestus*. Heredity (Edinb). 2020;124(5):621–32. 32157181 10.1038/s41437-020-0304-1 PMC7171194

[49] Pennacchio LA, et al. Enhancers: five essential questions. Nat Rev Genet. 2013;14(4):288–95. 23503198 10.1038/nrg3458 PMC4445073

[50] Ingham VA, et al. The transcription factor Maf-S regulates metabolic resistance to insecticides in the malaria vector *Anopheles gambiae*. BMC Genomics. 2017;18(1):669. 28854876 10.1186/s12864-017-4086-7 PMC5577768

[51] Amenya DA, et al. Over expression of a cytochrome P450 (CYP6P9) in a major African malaria vector, *Anopheles funestus*, resistant to pyrethroids. Insect Mol Biol. 2008;17(1):19–25. 18237281 10.1111/j.1365-2583.2008.00776.x

[52] Wondji CS, et al. Two duplicated P450 genes are associated with pyrethroid resistance in *Anopheles funestus*, a major malaria vector. Genome Res. 2009;19(3):452–9. 19196725 10.1101/gr.087916.108 PMC2661802

[53] Vontas J, et al. Rapid selection of a pyrethroid metabolic enzyme CYP9K1 by operational malaria control activities. Proc Natl Acad Sci U S A. 2018;115(18):4619–24. 29674455 10.1073/pnas.1719663115 PMC5939083

[54] Wondji CS, et al. Impact of pyrethroid resistance on operational malaria control in Malawi. Proc Natl Acad Sci U S A. 2012;109(47):19063–70. 23118337 10.1073/pnas.1217229109 PMC3511128

[55] Wamba ANR, et al. The cytochrome P450 CYP325A is a major driver of pyrethroid resistance in the major malaria vector *Anopheles funestus* in Central Africa. Insect Biochem Mol Biol. 2021;138: 103647. 34530119 10.1016/j.ibmb.2021.103647

