# Supplementary material for: The duplicated cytochrome P450 CYP6P9a/b confers cross-resistance to a mitochondrial complex I inhibitor in the African malaria vector Anopheles funestus
Source: BMC Genomics. 2025 Sep 26;26:837. doi: 10.1186/s12864-025-11984-1 (PMC12465288; doi:10.1186/s12864-025-11984-1)
Supplement: Supplementary file 8 — Supplementary Material 8 [file 12864_2025_11984_MOESM8_ESM.docx]

**Supplementary data**

**Supplementary 1:** CDC Bottle bioassays

**Supplementary 1a:** Sample generation for bottle bioassay

|  |  | **No of Alive** | | **No of Dead** | | **Target**  **(Alive: Dead)** | **% Mortality (Corrected)** | |
| --- | --- | --- | --- | --- | --- | --- | --- | --- |
|  | **Time of exposure** | **30 mins.** | **60 mins.** | **30 mins.** | **60 mins.** |  | **30 mins.** | **60 mins** |
| **FUMOZ-R/FANG F_4_ hybrids** | **Negative control** | 86 | 216 | 4 | 15 | 100 alive | 0 % | 0% |
|  | **Permethrin control** | 51 | 49 | 146 | 653 | 100: 60 | 73±6.2% | 92±1.1% |
|  | **Sherlock 1xDC** | 61 | 42 | 132 | 576 | 100: 60 | 67±4.6% | 93±0.8% |
|  | **Sherlock 5xDC** | 42 | 20 | 175 | 646 | 100: 60 | 80±5.1% | 97±0.97% |
| **Mibellon F1** | **Negative control** | 75 | 90 | 2 | 5 | 100 alive | 2.6±1.6% | 0% |
|  | **Permethrin control** | 94 | 66 | 39 | 87 | 100:60 | 29.3±9.6% | 54.5±7.2% |
|  | **Sherlock 1xDC** | 32 | 3 | 247 | 149 | 100:60 | 88.5±4.1% | 97.9±0.9% |
|  | **Sherlock 5xDC** | 27 | 3 | 324 | 178 | 100:60 | 92.3±1.5% | 98.3±1.1% |

**Supplementary 1b:** Genotypic frequencies and allele frequencies of the 6.5 kb *SV* among FUMOZ-R/FANG F_4_ hybrids exposed to Permethrin bottles (A and B, respectively), Sherlock 1xDC (C and D, respectively), and Sherlock 5xDC (E and F, respectively)

**B**

**A**

**C**

**D**

**F**

**E**

**Supplementary 1c:** Genotypic and allelic frequencies of *CYP6P9a* among FUMOZ-R/FANG F_4_ hybrids exposed to Permethrin (A and B, respectively), Sherlock 1xDC (C and D, respectively), and Sherlock 5xDC (E and F, respectively)

**A**

**B**


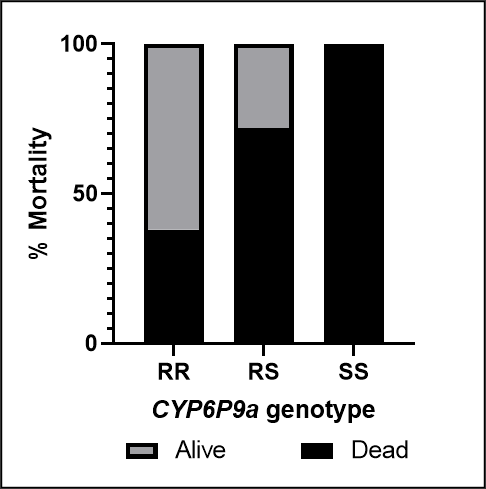


**C**

**D**

**E**

**F**

**Supplementary 1d:** Genotypic and allelic frequencies of *CYP6P9b* among FUMOZ-R/FANG F_4_ hybrids exposed to Permethrin (A and B, respectively), Sherlock 1xDC (C and D, respectively), and Sherlock 5xDC (E and F, respectively)

**B**

**A**

**C**

**D**

**F**

**E**

**Supplementary 1e:** The association between the combined three markers and the ability of FUMOZ-R/FANG F_4_ hybrids to survive exposure to insecticide bottles. The correlation between the combined three markers and the ability of F_4_ hybrids to survive permethrin, 1xDC Sherlock and 5xDC Sherlock bottles

|  | **SV/CYP6P9a/CYP6P9b** | **OR** | **CI** | **p value** |
| --- | --- | --- | --- | --- |
| **Permethrin bottles** | **SV+/SV+/RR/RR vs SV-/SV-/SS/SS** | 38.36 | 17.05 to 79.72 | <0.0001 |
|  | **SV+/SV+/RR/RR vs SV-/SV-/RS/SS** | Infinity | 145.8 to Infinity | <0.0001 |
|  | **SV+/SV+/RR/RR vs SV+/SV-/RS/RS** | 37.92 | 16.84 to 78.85 | <0.0001 |
|  | **SV+/SV+/RR/RR vs SV+/SV+/RS/RS** | 11.51 | 5.84 to 22.91 | <0.0001 |
|  | **SV+/SV+/RR/RR vs SV+/SV+/RR/RS** | 0 | 0 to 0.23 | <0.0001 |
|  | **SV+/SV+/RR/RS vs SV-/SV-/SS/SS** | Infinity | 169.6 to Infinity | <0.0001 |
|  | **SV+/SV+/RR/RS vs SV-/SV-/RS/SS** | Infinity | 1980 to Infinity | <0.0001 |
|  | **SV+/SV+/RR/RS vs SV+/SV-/RS/RS** | Infinity | 167.6 to Infinity | <0.0001 |
|  | **SV+/SV+/RR/RS vs SV+/SV+/RS/RS** | Infinity | 57.58 to infinity | <0.0001 |
|  | **SV+/SV+/RS/RS vs SV-/SV-/SS/SS** | 3.334 | 1.596 to 6.56 | 0.0008 |
|  | **SV+/SV+/RS/RS vs SV-/SV-/RS/SS** | Infinity | 14.07 to Infinity | <0.0001 |
|  | **SV+/SV+/RS/RS vs SV+/SV-/RS/RS** | 3.296 | 1.58 to 6.48 | 0.0013 |
|  | **SV+/SV-/RS/RS vs SV-/SV-/SS/SS** | 1.011 | 0.44 to 2.32 | >0.9999 |
|  | **SV+/SV-/RS/RS vs SV-/SV-/RS/SS** | Infinity | 3.73 to Infinity | 0.0002 |
|  | **SV-/SV-/RS/SS vs SV-/SV-/SS/SS** | 0 | 0 to 0.27 | 0.0002 |
| **1x DC sherlock bottles** | **SV+/SV+/RR/RR vs SV-/SV-/SS/SS** | Infinity | 120.8 to Infinity | <0.0001 |
|  | **SV+/SV+/RR/RR vs SV+/SV-/RS/RS** | 20.75 | 9.93 to 41.25 | <0.0001 |
|  | **SV+/SV+/RR/RR vs SV+/SV+/RS/RS** | 6.039 | 3.13 to 11.6 | <0.0001 |
|  | **SV+/SV+/RR/RR vs SV+/SV+/RR/RS** | 3.037 | 1.6 to 5.83 | 0.001 |
|  | **SV+/SV+/RR/RS vs SV-/SV-/SS/SS** | Infinity | 43.06 to Infinity | <0.0001 |
|  | **SV+/SV+/RR/RS vs SV+/SV-/RS/RS** | 6.83 | 3.53 to 13.2 | <0.0001 |
|  | **SV+/SV+/RR/RS vs SV+/SV+/RS/RS** | 1.99 | 1.13 to 3.44 | 0.0233 |
|  | **SV+/SV+/RS/RS vs SV-/SV-/SS/SS** | Infinity | 21.79 to Infinity | <0.0001 |
|  | **SV+/SV+/RS/RS vs SV+/SV-/RS/RS** | 3.44 | 1.77 to 6.58 | 0.0002 |
|  | **SV+/SV-/RS/RS vs SV-/SV-/SS/SS** | Infinity | 5.87 to Infinity | <0.0001 |
| **5x DC sherlock bottles** | **SV+/SV+/RR/RR vs SV-/SV-/SS/SS** | Infinity | 1980 to Infinity | <0.0001 |
|  | **SV+/SV+/RR/RR vs SV-/SV-/RS/SS** | Infinity | 1980 to Infinity | <0.0001 |
|  | **SV+/SV+/RR/RR vs SV+/SV-/RS/RS** | Infinity | 119.2 to Infinity | <0.0001 |
|  | **SV+/SV+/RR/RR vs SV+/SV+/RS/RS** | Infinity | 1980 to Infinity | <0.0001 |
|  | **SV+/SV+/RR/RR vs SV+/SV+/RR/RS** |  |  | >0.9999 |
|  | **SV+/SV+/RR/RS vs SV-/SV-/SS/SS** | Infinity | 1980 to Infinity | <0.0001 |
|  | **SV+/SV+/RR/RS vs SV-/SV-/RS/SS** | Infinity | 1980 to Infinity | <0.0001 |
|  | **SV+/SV+/RR/RS vs SV+/SV-/RS/RS** | Infinity | 119.2 to Infinity | <0.0001 |
|  | **SV+/SV+/RR/RS vs SV+/SV+/RS/RS** | Infinity | 1980 to Infinity | <0.0001 |
|  | **SV+/SV+/RS/RS vs SV-/SV-/SS/SS** |  |  | >0.9999 |
|  | **SV+/SV+/RS/RS vs SV-/SV-/RS/SS** |  |  | >0.9999 |
|  | **SV+/SV+/RS/RS vs SV+/SV-/RS/RS** | 0 | 0 to 0.17 | <0.0001 |
|  | **SV+/SV-/RS/RS vs SV-/SV-/SS/SS** | Infinity | 5.94 to Infinity | <0.0001 |
|  | **SV+/SV-/RS/RS vs SV-/SV-/RS/SS** | Infinity | 5.94 to Infinity | <0.0001 |
|  | **SV-/SV-/RS/SS vs SV-/SV-/SS/SS** |  |  | >0.9999 |

**Supplementary 2: WHO cone bioassays**

**Supplementary 2a:** Genotypic and allelic of the L119F_GSTe2 frequencies among *An. funestus* F_1_ Mibellon strains exposed to PermaNet 2.0 (A and B, respectively) and 0.7% incorporated Sherlock net (C and D, respectively)

**B**

**A**

**C**

**D**

**Supplementary 2b:** Table of significance showing the genotype and allele frequencies of the L119F-*GSTe2* pyrethroid-resistance marker in field *An. funestus* strains

|  |  | **OR** | **CI** | **p value** | **Summary** |
| --- | --- | --- | --- | --- | --- |
| **PermaNet 2.0** | **RR vs SS** | Infinity | 1.957 to Infinity | 0.0059 | ** |
|  | **RR vs RS** | Infinity | 1.490 to Infinity | 0.0159 | * |
|  | **RS vs SS** | 1.225 | 0.6713 to 2.249 | 0.5454 | ns |
|  | **R vs S** | 1.658 | 0.8289 to 3.304 | 0.2156 | ns |
| **0.7% incorporated Sherlock net** | **RR vs SS** | Infinity | 0.9918 to Infinity | 0.0901 | ns |
|  | **RR vs RS** | Infinity | 2.537 to Infinity | 0.0022 | ** |
|  | **RS vs SS** | 0.4085 | 0.2319 to 0.7280 | 0.0033 | ** |
|  | **R vs S** | 0.7619 | 0.4164 to 1.372 | 0.452 | ns |

**Supplementary 2c:** The combined effect of the three markers on the ability of FUMOZ-R/FANG F_4_ hybrids to survive exposure to bed nets. The correlation between the combined three markers and the ability of FUMOZ-R/FANG F_4_ hybrids to survive exposure to PermaNet 2.0 and Sherlock bed net

|  | **SV/CYP6P9a/CYP6P9b** | **OR** | **CI** | **p value** |
| --- | --- | --- | --- | --- |
| **PermaNet 2.0** | **SV+/SV+/RR/RR vs SV-/SV-/SS/SS** | Infinity | 1980 to Infinity | <0.0001 |
|  | **SV+/SV+/RR/RR vs SV+/SV-/SS/SS** | Infinity | 28.91 to Infinity | <0.0001 |
|  | **SV+/SV+/RR/RR vs SV+/SV-/RS/RS** |  |  | >0.9999 |
|  | **SV+/SV+/RR/RR vs SV+/SV+/RS/RS** |  |  | >0.9999 |
|  | **SV+/SV+/RR/RR vs SV+/SV+/RS/RR** | Infinity | 9.31 to Infinity | <0.0001 |
|  | **SV+/SV+/RS/RR vs SV-/SV-/SS/SS** | Infinity | 82.96 to Infinity | <0.0001 |
|  | **SV+/SV+/RS/RR vs SV+/SV-/SS/SS** | 3 | 1.67 to 5.39 | 0.0004 |
|  | **SV+/SV+/RS/RR vs SV+/SV-/RS/RS** | 0 | 0 to 0.11 | <0.0001 |
|  | **SV+/SV+/RS/RR vs SV+/SV+/RS/RS** | 0 | 0 to 0.11 | <0.0001 |
|  | **SV+/SV+/RS/RS vs SV-/SV-/SS/SS** | Infinity | 1980 to Infinity | <0.0001 |
|  | **SV+/SV+/RS/RS vs SV+/SV-/SS/SS** | Infinity | 28.91 to Infinity | <0.0001 |
|  | **SV+/SV+/RS/RS vs SV+/SV-/RS/RS** |  |  | >0.9999 |
|  | **SV+/SV-/RS/RS vs SV-/SV-/SS/SS** | Infinity | 1980 to Infinity | <0.0001 |
|  | **SV+/SV-/RS/RS vs SV+/SV-/SS/SS** | Infinity | 28.91 to Infinity | <0.0001 |
|  | **SV-+/SV-/SS/SS vs SV-/SV-/SS/SS** | Infinity | 28.91 to Infinity | <0.0001 |
| **0.7% incorporated Sherlock Net** | **SV+/SV+/RR/RR vs SV-/SV-/SS/SS** | Infinity | 156.1 to Infinity | <0.0001 |
|  | **SV+/SV+/RR/RR vs SV-/SV-/RS/SS** | Infinity | 57.58 to Infinity | <0.0001 |
|  | **SV+/SV+/RR/RR vs SV+/SV-/RS/RS** | Infinity | 68.71 to Infinity | <0.0001 |
|  | **SV+/SV+/RR/RR vs SV+/SV+/RR/RS** | Infinity | 5.42 to Infinity | <0.0001 |
|  | **SV+/SV+/RR/RS vs SV-/SV-/SS/SS** | 29.99 | 14.02 to 65.09 | <0.0001 |
|  | **SV+/SV+/RR/RS vs SV-/SV-/RS/SS** | 9.91 | 4.96 to 18.59 | <0.0001 |
|  | **SV+/SV+/RR/RS vs SV+/SV-/RS/RS** | 11.95 | 6.17 to 22.86 | <0.0001 |
|  | **SV+/SV+/RR/RS vs SV+/SV+/RS/RS** | 0.2034 | 0.07 to 0.58 | 0.0046 |
|  | **SV+/SV+/RS/RS vs SV-/SV-/SS/SS** | 147.4 | 46.52 to 402.5 | <0.0001 |
|  | **SV+/SV+/RS/RS vs SV-/SV-/RS/SS** | 48.73 | 16.46 to 130.1 | <0.0001 |
|  | **SV+/SV+/RS/RS vs SV+/SV-/RS/RS** | 58.76 | 19.56 to 157.1 | <0.0001 |
|  | **SV+/SV-/RS/RS vs SV-/SV-/SS/SS** | 2.509 | 1.27 to 5.24 | 0.0153 |
|  | **SV+/SV-/RS/RS vs SV-/SV-/RS/SS** | 0.8293 | 0.45 to 1.53 | 0.6467 |
|  | **SV-/SV-/RS/SS vs SV-/SV-/SS/SS** | 3.026 | 1.48 to 6.22 | 0.0025 |

**Supplementary 3:** WHO tunnel tests

**Supplementary 3a:** Genotypic and allelic of the 6.5 kb *SV* frequencies among FUMOZ-R/FANG F_4_ hybrids exposed to PermaNet 2.0: (Mortality (A and B, respectively) and blood-feeding status (C and D, respectively)

**B**

**A**

**C**

**DD**

**Supplementary 3b:** Genotypic and allelic of the 6.5 kb *SV* frequencies among FUMOZ-R/FANG F_4_ hybrids exposed to sherlock net: Mortality (C and D, respectively) and blood-feeding status (C and D, respectively)

**A**

**B**

**D**

**C**

**Supplementary 3c:** Genotypic and allelic of the *CYP6P9a* frequencies among FUMOZ-R/FANG F_4_ hybrids exposed to PermaNet 2.0: Mortality (C and D, respectively) and blood-feeding status (C and D, respectively)

**B**

**A**

**C**

**D**

**Supplementary 3d:** Genotypic and allelic of the *CYP6P9a* frequencies among F_4_ hybrids exposed to 0.7% incorporated Sherlock net: Mortality (C and D, respectively) and blood-feeding status (C and D, respectively)

**B**

**A**

**C**

**D**

**Supplementary 3e:** Genotypic and allelic of the *CYP6P9b* frequencies among FUMOZ-R/FANG F_4_ hybrids exposed to PermaNet 2.0: Mortality (C and D, respectively) and blood-feeding status (C and D, respectively)

**B**

**A**

**C**

**D**

**Supplementary 3f:** Genotypic and allelic of the *CYP6P9b* frequencies among FUMOZ-R/FANG F_4_ hybrids exposed to 0.7% incorporated Sherlock net: Mortality (C and D, respectively) and blood-feeding status (C and D, respectively)

**B**

**A**

**C**

**D**

**Supplementary 3g:** The ability of FUMOZ-R/FANG F_4_ hybrids with or without resistant markers to survive after successfully penetrating through bed net and blood-feeding on bait. Genotypic frequencies of *CYP6P9a* and *CYP6P9b* among FUMOZ-R/FANG F_4_ hybrids exposed to PermaNet 2.0 and 0.7% incorporated Sherlock bed net

**Supplementary 3h:** Correlation between genotypes of the 6.5 kb SV, *CYP6P9a* and *CYP6P9b* and the ability of FUMOZ-R/FANG F_4_ hybrids to survive exposure to 0.7% incorporated Sherlock net and PermaNet 2.0 post blood feeding (For the Sherlock-exposed comparison, no SS individuals for the structural variant were detected in either the alive or dead blood-fed groups, explaining the empty cells; all hybrids with the SS genotype were exclusively in the unfed group)

|  |  | **OR** | **CI** | **P value** | **OR** | **CI** | **P value** | **OR** | **CI** | **P value** |
| --- | --- | --- | --- | --- | --- | --- | --- | --- | --- | --- |
|  |  | ***6.5 kb* SV** | | | ***CYP6P9a*** | | | ***CYP6P9b*** | | |
| **Permanent 2.0** | **RR vs SS** | 13.67 | 6.76-27.17 | <0.0001 | 46 | 18.52-105.0 | <0.0001 | 3.48 | 1.91-6.22 | <0.0001 |
|  | **RS vs SS** | 5.03 | 2.55-10.12 | <0.0001 | 4.33 | 2.31-7.92 | <0.0001 | 3.96 | 2.16-6.94 | <0.0001 |
|  | **RR vs RS** | 2.72 | 1.5-4.9 | 0.001 | 10.6 | 4.72-22.53 | <0.0001 | 0.88 | 0.50-1.53 | 0.7703 |
| **0.7% incorporated Sherlock** | **RR vs SS** | - | - | - | 19.5 | 9.25-38.48 | <0.0001 | 29.33 | 13.59-61.66 | <0.0001 |
|  | **RS vs SS** | - | - | - | 5.03 | 2.55-10.12 | 0.0177 | 2.25 | 1.21-4.17 | 0.0177 |
|  | **RR vs RS** | 1 | 0.57-1.76 | >0.9999 | 3.9 | 2.14-7.08 | <0.0001 | 13.04 | 6.25-26.61 | <0.0001 |

**Supplementary 3i:** The combined additive advantage of the three markers on the ability of FUMOZ-R/FANG F_4_ hybrids to survive exposure to bed nets

|  | 6.5kb SV/CYP6P9a/CYP6P9b | **OR** | **CI** | **p value** |
| --- | --- | --- | --- | --- |
| **PermaNet 2.0** | **SV+/SV+/RR/RR vs SV-/SV-/RS/RS** | Infinity | 1980 to Infinity | <0.0001 |
|  | **SV+/SV+/RR/RR vs SV+/SV-/SS/RS** | Infinity | 33.88 to Infinity | <0.0001 |
|  | **SV+/SV+/RR/RR vs SV+/SV-/RS/RS** | Infinity | 14.07 to Infinity | <0.0001 |
|  | **SV+/SV+/RR/RR vs SV+/SV+/RR/RS** | Infinity | 4.14 to Infinity | <0.0001 |
|  | **SV+/SV+/RR/RS vs SV-/SV-/RS/RS** | Infinity | 156.1 to Infinity | <0.0001 |
|  | **SV+/SV+/RR/RS vs SV+/SV-/SS/RS** | **3.026** | 1.48 to 6.22 | 0.0025 |
|  | **SV+/SV+/RR/RS vs SV+/SV-/RS/RS** | 7.2 | 3.68 to 14.53 | <0.0001 |
|  | **SV+/SV-/RS/RS vs SV-/SV-/RS/RS** | Infinity | 57.58 to Infinity | <0.0001 |
|  | **SV+/SV-/RS/RS vs SV+/SV-/SS/RS** | 2.38 | 1.33 to 4.23 | 0.0042 |
|  | **SV+/SV-/SS/RS vs SV-/SV-/RS/RS** | Infinity | 24.63 to Infinity | <0.0001 |
| **0.7% incorporated Sherlock bed net** | **SV+/SV+/RR/RR vs SV-/SV-/SS/SS** | Inf. | 65.7 to Inf. | <0.0001 |
|  | **SV+/SV+/RR/RR vs SV-/SV-/RS/RS** | Inf. | 64.7 to Inf. | <0.0001 |
|  | **SV+/SV+/RR/RR vs SV+/SV+/SS/RR** | Inf. | 64.7 to Inf. | <0.0001 |
|  | **SV+/SV+/RR/RR vs SV+/SV+/RS/SS** | Inf. | 64.7 to Inf. | <0.0001 |
|  | **SV+/SV+/RR/RR vs SV+/SV-/RS/RR** | 0.44 | 0.23 to 0.89 | 0.028 |
|  | **SV+/SV+/RR/RR vs SV+/SV-/RS/RS** | 4.21 | 2.3 to 7.5 | <0.0001 |
|  | **SV+/SV+/RR/RR vs SV+/SV+/RR/RS** | 2.3 | 1.3 to 4.2 | 0.0059 |
|  | **SV+/SV+/RR/RS vs SV-/SV-/SS/SS** | Inf. | 28.9 to Inf. | <0.0001 |
|  | **SV+/SV+/RR/RS vs SV-/SV-/RS/RS** | Inf. | 28.9 to Inf. | <0.0001 |
|  | **SV+/SV+/RR/RS vs SV+/SV+/SS/RR** | Inf. | 28.9 to Inf. | <0.0001 |
|  | **SV+/SV+/RR/RS vs SV+/SV+/RS/SS** | Inf. | 28.9 to Inf. | <0.0001 |
|  | **SV+/SV+/RR/RS vs SV+/SV-/RS/RR** | 0.19 | 0.1 to 0.4 | <0.0001 |
|  | **SV+/SV+/RR/RS vs SV+/SV-/RS/RS** | 1.83 | 1.0 to 3.2 | 0.0449 |
|  | **SV+/SV-/RS/RS vs SV-/SV-/SS/SS** | Inf. | 15.7 to Inf. | <0.0001 |
|  | **SV+/SV-/RS/RS vs SV-/SV-/RS/RS** | Inf. | 15.7 to Inf. | <0.0001 |
|  | **SV+/SV-/RS/RS vs SV+/SV+/SS/RR** | Inf. | 15.7 to Inf, | <0.0001 |
|  | **SV+/SV-/RS/RS vs SV+/SV+/RS/SS** | Inf. | 15.7 to Inf. | <0.0001 |
|  | **SV+/SV-/RS/RS vs SV+/SV-/RS/RR** | 0.11 | 0.1 to 0.2 | <0.0001 |

**Supplementary 3j:** The combined effect of the three markers on the ability of FUMOZ-R/FANG F_4_ hybrids to survive after successfully penetrating through bed net and blood feeding. Genotypic frequencies of the combined three markers among FUMOZ-R/FANG F_4_ hybrids exposed to PermaNet 2.0 and Sherlock bed net

|  | 6.5kb SV/CYP6P9a/CYP6P9b | **OR** | **CI** | **p value** |
| --- | --- | --- | --- | --- |
| **PermaNet 2.0** | **SV+/SV+/RR/RR vs SV-/SV-/RS/RS** | Infinity | 1980 to Infinity | <0.0001 |
|  | **SV+/SV+/RR/RR vs SV+/SV-/SS/RS** | Infinity | 1980 to Infinity | <0.0001 |
|  | **SV+/SV+/RR/RR vs SV+/SV+/RS/SS** | Infinity | 1980 to Infinity | <0.0001 |
|  | **SV+/SV+/RR/RR vs SV+/SV-/RS/RS** | Infinity | 14.07 to Infinity | <0.0001 |
|  | **SV+/SV+/RR/RR vs SV+/SV+/RR/RS** | Infinity | 12.16 to Infinity | <0.0001 |
|  | **SV+/SV+/RR/RS vs SV-/SV-/RS/RS** | Infinity | 65.68 to Infinity | <0.0001 |
|  | **SV+/SV+/RR/RS vs SV+/SV-/SS/RS** | Infinity | 65.68 to Infinity | <0.0001 |
|  | **SV+/SV+/RR/RS vs SV+/SV+/RS/SS** | Infinity | 65.68 to Infinity | <0.0001 |
|  | **SV+/SV+/RR/RS vs SV+/SV-/RS/RS** | 1.15 | 0.64 to 2.12 | 0.7609 |
|  | **SV+/SV-/RS/RS vs SV-/SV-/RS/RS** | Infinity | 57.58 to Infinity | <0.0001 |
|  | **SV+/SV-/RS/RS vs SV+/SV-/SS/RS** | Infinity | 57.58 to Infinity | <0.0001 |
|  | **SV+/SV-/RS/RS vs SV+/SV+/RS/SS** | Infinity | 57.58 to Infinity | <0.0001 |
|  | **SV+/SV+/RS/SS vs SV-/SV-/RS/RS** |  |  | >0.9999 |
|  | **SV+/SV+/RS/SS vs SV+/SV-/SS/RS** |  |  | >0.9999 |
|  | **SV+/SV-/SS/RS vs SV-/SV-/RS/RS** |  |  | >0.9999 |
| **0.7% incorporated Sherlock bed net** | **SV+/SV+/RR/RR vs SV+/SV+/SS/SS** | Infinity | 195.6 to Infinity | <0.0001 |
|  | **SV+/SV+/RR/RR vs SV+/SV+/RS/SS** | 16.43 | 7.59 to 34.98 | <0.0001 |
|  | **SV+/SV+/RR/RR vs SV+/SV-/RS/RR** | 10.3 | 4.86 to 22.16 | <0.0001 |
|  | **SV+/SV+/RR/RR vs SV+/SV-/RS/RS** | 12.14 | 5.71 to 26.26 | <0.0001 |
|  | **SV+/SV+/RR/RR vs SV+/SV+/SS/RR** | 0 | 0 to 0.28 | 0.0007 |
|  | **SV+/SV+/RR/RR vs SV+/SV+/RR/RS** | 5.89 | 2.51 to 11.66 | <0.0001 |
|  | **SV+/SV+/RR/RS vs SV+/SV+/SS/SS** | Infinity | 43.06 to Infinity | <0.0001 |
|  | **SV+/SV+/RR/RS vs SV+/SV+/RS/SS** | 3.045 | 1.69 to 5.47 | 0.0002 |
|  | **SV+/SV+/RR/RS vs SV+/SV-/RS/RR** | 1.909 | 1.08 to 3.29 | 0.0335 |
|  | **SV+/SV+/RR/RS vs SV+/SV-/RS/RS** | 2.25 | 1.27 to 3.92 | 0.0071 |
|  | **SV+/SV+/RR/RS vs SV+/SV+/SS/RR** | 0 | 0 to 0.05 | <0.0001 |
|  | **SV+/SV+/SS/RR vs SV+/SV+/SS/SS** | infinity | 1980 to Infinity | <0.0001 |
|  | **SV+/SV+/SS/RR vs SV+/SV+/RS/SS** | Infinity | 57.58 to Infinity | <0.0001 |
|  | **SV+/SV+/SS/RR vs SV+/SV-/RS/RR** | Infinity | 36.68 to Infinity | <0.0001 |
|  | **SV+/SV+/SS/RR vs SV+/SV-/RS/RS** | Infinity | 43.06 to Infinity | <0.0001 |
|  | **SV+/SV-/RS/RS vs SV+/SV+/SS/SS** | Infinity | 19.22 to Infinity | <0.0001 |
|  | **SV+/SV-/RS/RS vs SV+/SV+/RS/SS** | 1.354 | 0.77 to 2.4 | 0.3782 |
|  | **SV+/SV-/RS/RS vs SV+/SV-/RS/RR** | 0.85 | 0.49 to 1.51 | 0.6675 |
|  | **SV+/SV-/RS/RR vs SV+/SV+/SS/SS** | Infinity | 22.7 to Infinity | <0.0001 |
|  | **SV+/SV-/RS/RR vs SV+/SV+/RS/SS** | 1.595 | 0.88 to 2.82 | 0.146 |
|  | **SV+/SV+/RS/SS vs SV+/SV+/SS/SS** | Infinity | 14.07 to Infinity | <0.0001 |
